# Investigating the Supramolecular Assemblies under the Confinement in Hybrid Mesoporous Silica Films

**DOI:** 10.1002/smsc.202500369

**Published:** 2025-08-17

**Authors:** Jakub Kusz, Cédric Boissiere, Michel Wong Chi Man, Tangui Le Bahers, Clément Sanchez, Stephane Parola

**Affiliations:** ^1^ École Normale Supérieure de Lyon CNRS Université Claude Bernard Lyon 1 Laboratoire de Chimie UMR 5182 46 Allée d’Italie 69364 Lyon France; ^2^ Colloid Chemistry Department Max Planck Institute of Colloids and Interfaces 14476 Potsdam Germany; ^3^ Laboratoire de Chimie de la Matière Condensée de Paris (LCMCP) Sorbonne Université CNRS Collège de France UMR 7574 4 place Jussieu 75005 Paris France; ^4^ Institut Charles Gerhardt Montpellier Univ. Montpellier CNRS ENSCM 34293 Montpellier France

**Keywords:** confinement, films, hybrid, hydrogen bonds, mesoporous, silica, supramolecular

## Abstract

This study investigates hydrogen‐bond‐mediated supramolecular assemblies within the confined environment of hybrid mesoporous silica films. Infrared spectroscopy is used to examine hydrogen bonding (HB) between ureidophenyl moieties introduced into the silica framework via the co‐condensation between tetraethoxysilane and *N*‐Phenyl‐*N*‐[3‐(triethoxysilyl)propyl]urea (PhU‐TES) precursor. Shifts in the amide I and amide II bands serve as indicators of HB cleavage or formation. These inter actions are initially restricted by the presence of the surfactant, mirroring effects previously observed for pyrene excimer formation and suggesting a broader principle in supramolecular assemblies in hybrid mesostructured materials. Three distinct ureido nanoenvironments can be identified within the mesopores: ureido–ureido molecular assemblies, ureido–silanol architectures, and free, nonbonded ureido moieties. The relative abundance of these environments depends on precursor concentration: higher loading promotes ureido–ureido interactions, while lower loading favors ureido–silanol bonding. In ternary systems, bromoalkyl groups partially hinder HB, although complete suppression is not achieved, indicating potential spatial segregation within the pores. These findings highlight the complex role of weak interactions in shaping supramolecular architectures within hybrid mesoporous materials.

## Graphical abstract

Different molecular environments can be found in hybrid mesoporous silica films. Ureido moieties form supramolecular assemblies via hydrogen bonds, bond with silanols at the pore interface, or remain separated by surfactant molecules. The relative abundance of these environments can be probed by infrared spectroscopy and controlled by molecular design.

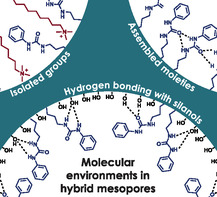

## 1 Introduction

In confined environments, the properties and behavior of matter at the molecular level differ significantly from those observed in the bulk phase.^[^ ^1^, ^2^, ^3^ ^]^ Such confinement can be used to control self‐assembly processes^[^ ^4^ ^]^ and influence reactivity and selectivity.^[^ ^5^ ^]^ Consequently, significant efforts have been directed toward understanding the mechanisms beneath these effects and developing synthetic strategies to tune the properties and performance of catalysts, sensors, membranes, and purification systems.

On the other hand, spatial confinement may also induce the organization of molecules into supramolecular assemblies.^[^ ^6^ ^]^ The recent surge in supramolecular chemistry has enabled the design of increasingly sophisticated systems composed of multiple components held together by weak, noncovalent interactions. Supramolecular organization under confinement has been extensively studied in biomolecular systems, such as the self‐assembly of peptides and amino acids, mimicking processes observed in nature—for instance, under the confinement of cellular environments.^[^ ^7^, ^8^, ^9^, ^10^ ^]^

Supramolecular chemistry at interfaces^[^ ^11^, ^12^, ^13^ ^]^ or within mesopores is particularly promising, offering new possibilities for the synthesis of advanced functional materials. Thanks to their sufficiently large pore diameters, mesoporous materials can serve as hosts for supramolecular or coordination‐based systems.^[^ ^14^ ^]^ Confinement within these cavities can substantially modify the properties of supramolecular structures by altering their interactions, improving dispersion, and enhancing material performance.^[^ ^15^, ^16^, ^17^ ^]^

Confinement imposed by mesoporous scaffolds has been utilized to control supramolecular aggregate self‐assembly in nanocages,^[^ ^18^ ^]^ grow supramolecular stacks of triarylamine for functional electrode fabrication,^[^ ^19^ ^]^ enhance self‐assembly of liquid crystals,^[^ ^20^ ^]^ stabilize the supramolecular structures,^[^ ^2^ ^]^ or construct of porphyrin‐functionalized metallacycles.^[^ ^16^ ^]^ In particular, coupling supramolecular chemistry with hybrid mesoporous silica has enabled in‐pore polymerization,^[^ ^21^, ^22^ ^]^ the development of intelligent and responsive materials,^[^ ^21^ ^]^ gated nanochemistry,^[^ ^23^, ^24^ ^]^ and stimuli‐responsive mesochannels or supramolecular nanovalves capable of controlling mass transport properties.^[^ ^23^, ^24^, ^25^, ^26^ ^]^

Functionalization of silica mesopores offers a strategy to tailor the confining environment through the choice of one or multiple organic groups, yielding multifunctional materials with enhanced properties compared to their monofunctional counterparts.^[^ ^27^, ^28^ ^]^ While the postfunctionalization approach (anchoring hybrid moieties onto surfacial silanols after the template removal) suffers from the lack of control over the spatial distribution of the functions within the material, an attractive alternative is the “one‐pot” method, in which all the silica precursors are introduced simultaneously. While it generally ensures more homogeneous distributions of functionalities within the matrix, its main side effect is the risk of entrapment of organic function within the silica wall, capable of decreasing the number of active sites or altering the mesostructure stability.^[^ ^29^, ^30^ ^]^

In most cases, the literature focuses on the final properties and performance of the hybrid materials but little attention was devoted to a better understanding of the species distribution and potential interactions between them, leading to the formation of supramolecular structures. When the organic moieties differ substantially in chemical nature, phase segregation or cluster formation may occur. Depending on the target application, such behavior could be either suppressed to preserve the synergistic effects of a bifunctional system or intentionally promoted to achieve controlled nanoscale segregation. Moreover, no such investigation has been devoted to thin films produced by the evaporation‐induced self‐assembly (EISA) method. The formation of these films is a dynamic process occurring on the timescale of minutes or even seconds,^[^ ^31^ ^]^ potentially impacting the spatial distribution of organic species and thus altering interfacial and confinement properties.

In our recent work, we explored how pyrene's excimer arising from π–π interactions can be formed within mesopores.^[^ ^32^ ^]^ These results suggest that similar self‐organization could arise from other types of weak forces, such as hydrogen bonds (HBs). These noncovalent interactions form when the electropositive hydrogen H is inserted between two electronegative atoms,^[^ ^33^ ^]^ and can be exploited in the formation of supramolecular systems^[^ ^12^ ^]^ or self‐assembled monolayers.^[^ ^34^, ^35^, ^36^, ^37^, ^38^, ^39^, ^40^ ^]^

Although the HB‐forming aminopropyl group has been relatively well studied, data are scarce concerning other functionalities, particularly derivatives of urea and thiourea. These groups also are known to form HB‐mediated supramolecular structures composed of organosilane precursors.^[^ ^41^, ^42^, ^43^, ^44^ ^]^ Understanding their behavior under confinement also holds practical significance, as H‐bonding hybrid materials are valuable for industrial applications in sorption^[^ ^45^, ^46^, ^47^, ^48^, ^49^, ^50^, ^51^, ^52^ ^]^ and sensing.^[^ ^53^, ^54^, ^55^ ^]^ A key advantage of the ureido group as a molecular probe is its ability to monitor HB formation via infrared (IR) spectroscopy. The amide I and amide II bands are particularly sensitive to HB interactions, typically exhibiting characteristic shifts in the IR spectrum, providing also insight into the bond strength.^[^ ^44^ ^]^

We propose that hydrogen bonding can serve as a model interaction to better understand the principles of hybrid self‐assembly. In this work, we aim to provide an analytical framework for experimental systems incorporating a single ureido‐functionalized precursor or coupling it with an apolar alkyl‐functionalized precursor via one‐pot synthesis. Our dual objectives are to identify factors that influence HB‐mediated supramolecular assembly and to determine the spatial organization of functional groups relative to each other and the silica framework in hybrid mesoporous thin films.

### 1.1 Choice of the Precursor

Numerous hybrid silica precursors containing ureido groups are commercially available or can be easily synthesized. For most of them, 3‐(trialkoxy)silyl group is linked through the propylene chain to the disubstituted ureido group that may be terminated with various functional groups. Therefore, the choice of the precursor should be based on the character of this terminal substituent.

Coupling the ureido moiety with an aromatic ring, such as a phenyl group, seems to be a promising perspective. Certain silica precursors bearing these two functionalities were shown to form self‐structured supramolecular assemblies thanks to the simultaneous presence of HBs (urea) and π–π stacking (phenyl).^[^ ^41^, ^56^, ^57^ ^]^ On the other hand, the applications of this type of precursor have been reported numerous times in the literature,^[^ ^58^, ^59^, ^60^ ^]^ particularly for the synthesis of bifunctional materials.^[^ ^27^, ^61^, ^62^ ^]^

An excellent candidate fulfilling these requirements is *N*‐Phenyl‐*N′*‐[3‐(triethoxysilyl)propyl]urea (PhU‐TES)—silica precursor bearing ureidopropyl group terminated with phenyl ring. Several works investigated the IR spectrum of materials based on PhU‐TES,^[^ ^60^, ^62^ ^]^ giving us an excellent starting point for analyzing hybrid mesoporous silica bearing this molecule. Even though it is not commercially available, its synthesis is straightforward and can be scaled up if needed.

## 2 Results and Discussion

### 2.1 Characterization of Phenylureido Precursor

**Figure 1:**
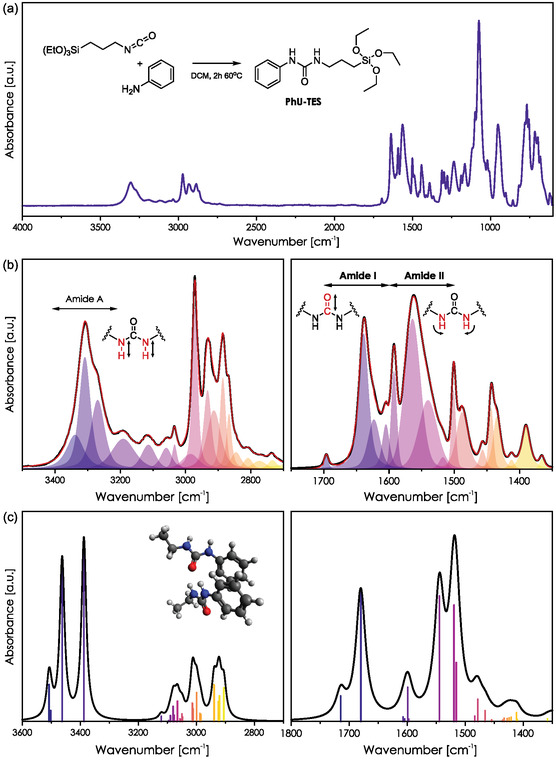
IR spectra of PhU‐TES precursor. a) Full spectrum in 4000–600 cm^−1^ range. b) Deconvolution of the amide A, amide I, and amide II regions. c) Spectra simulated by convoluting DFT computed harmonic frequencies with Gaussian functions having an FWHM of 5 cm^−1^.

PhU‐TES precursor was synthesized from isocyanatopropyltriethoxysilane and aniline (inset in **Figure** **1**a), as described in the experimental section. Figure 1a presents the Fourier‐transform infrared (FTIR) spectrum in the range of 4000–600 cm^−1^. The deconvolution of this spectrum within 3500–2700 and 1400–1800 cm^−1^ is shown in Figure 1b. Two intense signals around 1640 and 1550 cm^−1^ are commonly known as amide I and amide II bands.^[^ ^63^ ^]^ Their nature is widely described in the literature concerning the conformation studies of peptides and proteins.^[^ ^64^, ^65^, ^66^ ^]^ The vibrations in the amide I region, typically found around 1600–1700 cm^−1^, are primarily associated with the *μ*(C=O) stretching. Amide II region, located between 1500 and 1600 cm^−1^, originates mainly from the *δ*(N—H) bending and *μ*(C—N) stretching vibrations.

To assign the observed bands to their corresponding vibrational modes, we performed DFT calculations (Figure 1c; see Experimental Section for methodological details). Theoretical values obtained from simulations were cross‐validated with the literature data,^[^ ^44^, ^67^ ^]^ enabling confident assignment of the experimental bands to specific vibrations (Table S1, Supporting Information). The amide I region is dominated by a strong band localized at 1639 cm^−1^ corresponding to stretching of H‐bonded C=O. A weak band at 1696 cm^−1^ probably comes from a stretching of isolated C=O. Two more bands of moderate intensity within the amide I region are present at 1623 and 1605 cm^−1^. They can be assigned to *μ*(C=C) vibrations in the aromatic ring coupled to *δ*(C—H) bending. The same modes also result in a strong band within the amide II region at 1593 cm^−1^. The large signal of amide II present at 1550 cm^−1^ can be deconvoluted into three components at 1518, 1540, and 1563 cm^−1^, corresponding to several *δ*(N—H) bending modes coupled to in‐plane *μ*(C—C) and *δ*(C—H) in the aromatic ring. The band of moderate intensity localized at 1488 cm^−1^ stems from *δ*(N—H) vibrations, and a sharper band at 1501 cm^−1^ arises from in‐plane aryl *μ*(C=C) stretching coupled to *δ*(C—H) bending.

The zone between 3500 and 2600 cm^−1^, known as amide A, was deconvoluted similarly. It includes characteristic vibrations dominated by *μ*(N—H) stretching modes (here localized between 3400 and 3200 cm^−1^.). Several bands between 2800 and 3000 cm^−1^ correspond to *μ*(C—H) stretching vibrations in the aromatic ring and the short propyl chain.

The C=O and N—H vibrations in amide A, amide I, and amide II regions are highly sensitive to HB formation and cleavage. Downshifts of amide A and amide I are typically observed when HB is formed. On the contrary, amide II tends to upshift due to the H‐bonding. To affirm this effect in the investigated system, we must compare the spectra of solid PhU‐TES and PhU‐TES in a state that does not promote intermolecular PhU‐TES HB formation. The latter can be achieved by thermal treatment or by diluting the precursor with an aprotic solvent.^[^ ^44^, ^68^ ^]^ As thermal treatment leads to precursor degradation, PhU‐TES was dissolved in pure chloroform. Figure S1, Supporting Information, compares the spectrum of diluted precursor with the one obtained for the solid precursor. We observe a substantial evolution of the amide I band upon dilution. While the intensity of the initial signal localized at 1650 cm^−1^ decreases, the band of free *μ*(C=O) stretching grows around 1681 cm^−1^. Also, a slight redshift (Δ = 10 cm^−1^) is observed for the amide II band. Moreover, we see a slight upshift of the *μ*(C—C) band from 1593 to 1598 cm^−1^. On the other hand, the amide A band broadens and blueshifts from 3306 to 3332 cm^−1^. These evolutions of the IR spectra are characteristic of HB rupture.

Although the amide I and amide II vibrations appear to be perfect indicators of HB and could be used to study molecular interactions in hybrid mesoporous materials, the amide A band localized around 3300 cm^−1^ cannot be used for this purpose. Since surfacial silanol groups Si—OH and physisorbed water are present in the studied systems, broad *μ*(O—H) stretching vibration appears between 3700 and 3000 cm^−1^. Due to this overlap of *μ*(O—H) and amide A bands, the analysis of IR spectra will be limited to amide I and II regions,

### 2.2 Hybrid Films Derived from Phenylureido Precursor

**Figure 2:**
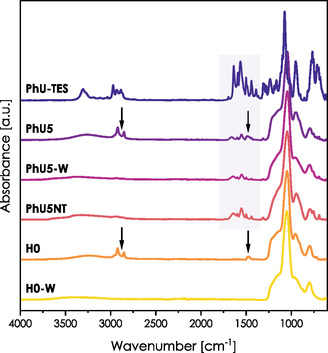
IR spectra of PhU‐TES precursor and thin films: PhU5 (hybrid raw), PhU5‐W (hybrid washed), and PhU5NT (hybrid nontemplated), H0 (nonhybrid raw), H0‐W (nonhybrid washed). Arrows indicate bands associated with the surfactant presence. The amide I and amide II regions are highlighted with a purple background.

A hybrid film structured with CTAB surfactant and containing 5 mol% of PhU‐TES was prepared by the co‐condensation method. As a reference, a nonhybrid film was synthesized using TEOS as the only silica source. These two films are denoted as PhU5 and H0, respectively. After the thermal stabilization of films at 80 °C, the templating agent (CTAB) was extracted by washing with pure ethanol, resulting in mesoporous samples PhU5‐W and H0‐W (where W accounts for the washed sample). Additionally, a hybrid nontemplated (nonmesoporous) film was synthesized from a similar sol containing no structure‐directing agent. This sample, denoted as PhU5NT, was only thermally stabilized, without any further treatment. We recorded the IR spectra of all samples by detaching a small piece of material from the substrate and placing it on the ATR prism (**Figure** **2**). IR spectra of unwashed and washed films were recorded under the same conditions and frequency range for all samples.

Before we discuss the amide I and II regions in the spectra of the hybrid film, several general points about mesoporous silica thin films must be addressed. All spectra presented in Figure 2 were normalized to the strongest vibration *μ*(Si—O—Si) localized at 1045 cm^−1^. The broad shoulder of this band between 1120 and 1250 cm^−1^ stems from several overlapping (Si—O—Si) antisymmetric stretching modes, while symmetric stretching is observed as a moderate signal at 800 cm^−1^. The band localized around 960 cm^−1^ corresponds to *μ*(Si—OH) stretching.^[^ ^69^, ^70^ ^]^ These vibrational bands of the inorganic framework are the same for hybrid and nonhybrid samples.

In the spectra of unwashed films, two well‐defined signals are present at 2850 and 2920 cm^−1^. They are attributed to *μ*(C—H) stretching vibrations of the CTAB chain. After extraction, these bands disappear, indicating a successful template removal. CTAB extraction is also confirmed by the suppression of the characteristic *δ*_sym_(N—CH3) signal localized typically between 1490 and 1465 cm^−1^.^[^ ^71^ ^]^

Here, two more changes in the spectrum can typically be stated (see Figure S2, Supporting Information): broadband between 3700 and 3000 cm^−1^ characteristic for stretching modes of the hydroxyl group of adsorbed water and the Si—OH species increases its intensity.^[^ ^69^, ^70^ ^]^ The presence of physically adsorbed water is also marked by a weak *δ*(H—O—H) bending band localized at 1635 cm^−1^. The broadening of the *μ*(O—H) and intensity increase of *δ*(H—O—H) bands are observed after the extraction. This suggests increased water adsorption due to better pore diffusion and a larger accessible surface area.

Comparing these results with the spectra of the PhU‐TES precursor, we see that the scissoring vibration of physisorbed water overlaps with the amide I band, which may make the analysis of this region ambiguous. Moreover, the physisorbed water may compete with other species in forming HBs with ureido groups.

This obstacle must be eliminated to obtain conclusive and reproducible data. As phenomena occurring at the surface of silica are in equilibrium with the environment, H_2_O can be removed by shifting this adsorption balance. Therefore, the sample was placed on the ATR prism and closed with a small cell filled with desiccant (drierite). Desorption of water occurs almost instantaneously, resulting in the complete suppression of *δ*(H—O—H) signal as shown in Figure S2, Supporting Information. The residual water can also be desorbed from unwashed samples thanks to the mass transport through the micropores. The same method was used to measure the IR spectra of hybrid samples before and after the template removal, ensuring no overlap in the amide I region.

All spectra in Figure 2 were recorded with this approach, which allowed us to avoid disturbance of the amide bands. Exactly as for the nonhybrid films, we observe here the disappearance of the CTAB fingerprint signals after the extraction. Thus, the effectiveness of washing is not disturbed by the organic functions present in the pores. In addition to the bands characteristic of the silica framework, we observe the PhU‐TES vibrations within the amide I and II regions, and a slight deformation of the broad *μ*(SiO—H) band due to overlap with the amide A.

**Figure 3:**
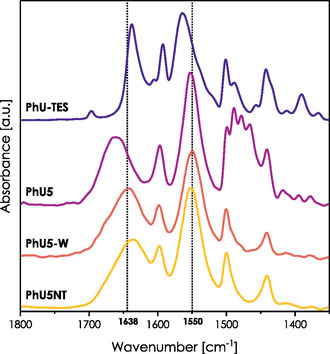
Evolution of the spectra within the amide I and II regions for hybrid films. A spectrum of pure PhU‐TES is given for reference. Dotted lines at 1638 and 1550 cm^−1^ correspond to the maximum of amide I and amide II bands in the PhU5‐W sample.

**Figure** **3** presents the amide I and II regions of hybrid films PhU5, PhU5‐W, and PhU5NT. The spectrum of the pure PhU‐TES precursor is also given for reference. Wavenumbers corresponding to the positions of the amide I and II bands are listed in **Table** **1**. The first thing that draws attention is the difference between the C=O vibrations of the solid PhU‐TES (1638 cm^−1^) and PhU5 film (1661 cm^−1^). This strong upshift of the amide I band suggests the change in the architecture of HB upon the film formation, and a downshift of amide II (1564 → 1552 cm^−1^) supports this hypothesis. The observed evolution of spectra corresponds to the cleavage of the HBs. We can also state a slight upshift of the *μ*(C—C) vibrations (1593 → 1597 cm^−1^), which is coherent with transitions observed for pure precursor dissolved in CHCl_3_ in Figure S1, Supporting Information.

**Table 1:** Description of hybrid samples, applied treatment methods, and obtained positions of amide I and II bands.

Sample	State	Amide I [cm^−1^]	Amide II [cm^−1^]
PhU5	Raw	1662	1552
PhU5‐W	Washed	1642	1551
PhU5NT	Nontemplated	1639	1552
PhU5+D_2_O	D_2_O vapors (in situ)	1653	Na
PhU5‐W+D_2_O	D_2_O vapors (in situ)	1625	Na
PhU5‐HMDS	HMDS vapors	1669	1537
PhU5‐TCMS	TCMS vapors	1664	1546
PhU5‐MTES	MTES co‐condensation	1654	1548

The initially bonded molecules probably separate during the evaporation‐induced self‐assembly and intercalate between the CTAB chains that form the micelles. Moreover, the strong broadening of the carbonyl stretching band indicates substantial heterogeneity of the molecular environment.

After the template removal, we observe a downshift of the amide I band from 1662 to 1642 cm^−1^, which suggests the reformation of bonds cleaved formerly. The lack of a significant shift in the amide II region may be associated with its lower sensitivity for HB formation, or rather indicate that these new bonds involve primarily the carbonyl groups of ureido moieties. The latter scenario is possible when ureido groups interact with other moieties able to form HB, such as silanol groups abundantly present at the silica surface.

Discussed spectra may be compared with a spectrum of nonporous film PhU5NT prepared without surfactant. For this sample, the amide I band is strongly downshifted—its peak is present at a wavenumber corresponding to (C=O) vibrations in pure PhU‐TES precursor (1638 cm^−1^). Moreover, we can note that in this case, the amide I band possesses some lower frequency components, which could signify the formation of HBs between the carbonyl group of the ureido function and surfacial silanol. DFT calculations showed us that, indeed, such a bond would be characterized by carbonyl vibrations present at a lower wavenumber (see **Table** **2**). In nontemplated material, hybrid species do not have the opportunity to reorganize upon the template removal, and they remain “frozen” within the inorganic structure. Therefore, the formation of HB with silanols would be promoted in contrast to mesoporous samples. These observations indicate that HBs must form at the early condensation stage and remain intact within the micelle (for mesoporous films) or in the inorganic silica matrix (for contemplated films).

**Table 2:** Vibration frequencies of *μ*(C=O) band for hybrid precursors in a free state and upon the formation of ureido–ureido (U—U) HB and ureido–silanol (U—SiOH) HB.

	Free U [cm^−1^]	U—U HB [cm^−1^]	U—SiOH HB [cm^−1^]
DFT	1724	1680 (Δ = 44)	1664 (Δ = 60)
Experimental	1675	1650 (Δ = 25)	1630 (Δ = 45)

**Figure 4:**
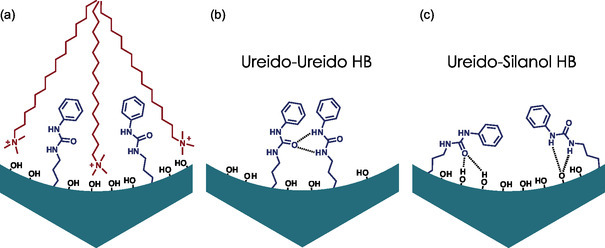
Molecular organization of PhU‐based hybrid films. a) Before the template removal, HB formation is restricted. b) After the template removal, HB may be formed between two ureido moieties, or c) between ureido and silanol groups.

Consecutive upshift and downshift of the amide I band imply the moderation of hydrogen bonding by the surfactant molecules as shown schematically in **Figure** **4**. Very similar behavior of hybrid systems based on fluorescent pyrene was reported by our group recently.^[^ ^32^ ^]^ In these systems, pyrene excimers were formed via π–π interactions, also after the removal of the structure‐directing agent. Therefore, despite the different probing species (ureido vs pyrene), different interaction types (HB vs π–π), and characterization technique (IR vs fluorescence spectroscopy), we can apply the same model in which the surfactant molecules moderate the weak interactions between the functional groups. While this suggests a more universal character of the proposed model, one significant difference between these two cases should be emphasized.

The π–π stacking of pyrene is a specific interaction and can occur only between two probe molecules. On the contrary, the HB can be formed not exclusively between two PhU moieties but also between PhU and surfacial silanol Si—OH. This behavior was observed by Chen et al. for PhU‐based mesoporous silica particles synthesized as catalysts for Diels–Alder reaction.^[^ ^62^ ^]^ The authors reported an important enhancement of catalytic performance and assigned it to the activation of catalytic centers (PhU groups) through the hydrogen bonding with silanols present at the pore surface. However, they did not consider the possibility of HB formation between two ureido moieties.

Hydrogen bonding between surfacial silanols and a probe also requires sufficient flexibility of the latter. Indeed, interactions between Si—OH and aminopropyl groups were investigated by Calvo et al..^[^ ^72^ ^]^ In the investigated system, the interactions were initially mediated by ion‐pair formation and subsequently led to HBs. Nevertheless, they showed that functional groups can bend easily to interact with the pore surface.

### 2.3 Investigation of Silanol–Ureido HBs

As the properties of the material will depend on the structure of the hybrid interface, it is important to assess the fractions of species that form ureido–ureido and ureido–silanol bonds. To discriminate between these two types of organizations, we can alter the chemistry of the interface in several ways.

One of the intrinsic characteristics of sol–gel silica is the presence of surface silanols, whose density depends on the synthesis and processing conditions but typically ranges between 0.7 and 1.9 groups per nm^−2^ for mesoporous silica.^[^ ^73^ ^]^ Reducing the Si—OH populations is usually achieved by temperature treatment, which leads to progressive condensation and the formation of new Si—O—Si bridges. However, this method cannot be applied to hybrid materials, as it would destroy the organic groups. Consequently, we must consider milder procedures such as hydrogen‐deuterium exchange or surface hydrophobization.

The first approach we explored is based on the substitution of silanol hydrogen by a deuterium atom. Such an exchange will lead to a downshift of all vibrations associated with the silanol moiety, including HBs formed with ureido species. It can be easily performed if a sample is put in contact with D_2_O vapors—e.g., in a closed cell on the ATR prism. Figure S3, Supporting Information, shows an example of deuteration on H0 and H0‐W films. As a result, the large *μ*(OH) band localized between 3600 and 2800 cm^−1^ is replaced by *μ*(OD) between 2750 and 2100 cm^−1^. The small residue present at the initial position is attributed to nonaccessible silanol groups. The band of *δ*(D—O—D) bending emerges at 1205 cm^−1^ and overlaps with *μ*(Si—O—Si) vibrations, as a result of heavy water physisorption.

Despite its theoretical potential, this approach may, however, be inadequate for systems containing ureido species, as the secondary exchange of NH into ND may occur (see Figure S4, Supporting Information). A shift of the amide I band is observed after removing the template from the deuterated sample (Figure S5, Supporting Information). We can state that ureido groups exhibit the same behavior in mesoporous systems, regardless of the presence of —OH or —OD species at the interface. While this exchange does not bring a clear answer about U—U versus SiOH—U H‐bonding, it may be developed into a very powerful method to probe the molecular organization of the material. The exchange kinetics would depend on the accessibility of hydrogen atoms^[^ ^74^ ^]^ and their potential interactions (e.g., H‐bonding). We observed that the exchange of ureido protons is not immediate (as for nonhybrid films) and progresses on a scale of several minutes (see Figure S6, Supporting Information).

**Figure 5:**
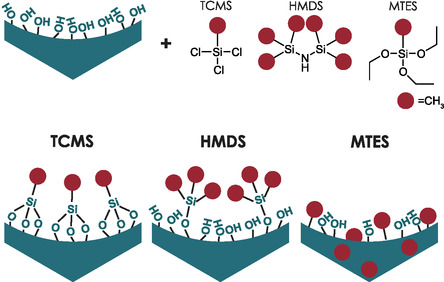
Three approaches toward surface hydrophobization and silanols substitution.

The second method that may lead us to understand the relationship between the ureido and silanol groups is surface functionalization, aiming to permanently reduce the number of available Si—OH moieties. We evaluate here three approaches (**Figure** **5**) that allow the substitution of Si—OH groups. 1) Postfunctionalization with hexamethyldisilazane (HMDS) is widely used for surface hydrophobization. Due to its high reactivity, the silanol groups can be effectively capped without the use of a catalyst. HMDS replaces a single silanol with a Si(CH_3_)_3_ group. 2) Postfunctionalization with trichloromethylsilane (TCMS) that acts similarly to HMDS and may be used as an alternative. The grafting results in the replacement of three silanols with one methyl group (even though it is fully effective only if one can condense the three Si—Cl functions with three Si—OH groups). 3) Co‐condensation with methyltriethoxysilane (MTES), which does not substitute any existing silanols but rather takes their place at the surface. Usually, high loading (up to 50%) and thermal treatment are necessary to completely hydrophobize the material as the methyl moieties tend to be also placed within the framework.^[^ ^75^ ^]^ This method also gives an interesting opportunity to “block” a large proportion of HB interactions at the hybrid interface already at the self‐assembly stage.

Each method leads to the decline of silanol density, but the differences in the surface chemistry may be substantial. Methyls grafted on the surface can act like little “pikes,” preventing ureido moieties from interacting with the surface. Moreover, additional steric effects in the case of HMDS functionalization should be anticipated.

Postfunctionalization was performed after the template removal by putting dry samples in contact with HMDS and TCMS vapors in a sealed chamber at 80 °C for 12 h. After the treatment, the films were washed with ethanol and dried. Functionalization with MTES was achieved by standard co‐condensation of TEOS, hybrid ureido precursor, and 20% of MTES. All three treatments were performed on H0 and PhU5 samples. Hydrophobized films are referred to as H0‐HMDS, H0‐TCMS, H0‐MTES, PhU5‐HMDS, PhU5‐TCMS, and PhU5‐MTES.

IR spectra of referential series H0 are presented in Figure S7, Supporting Information. Successful grafting is confirmed by the presence of characteristic bands at 844 and 1254 cm^−1^ corresponding to *ρ*(—CH_3_) rocking and *μ*(Si—CH_3_) stretching vibrations, respectively.^[^ ^76^, ^77^ ^]^ Additionally, we observe the reduction of (SiOH) vibration localized around 960 cm^−1^ for H0‐HMDS and H0‐TCMS films.

**Figure 6:**
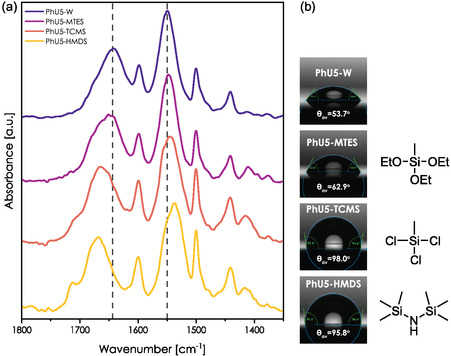
Effects of hydrophobization on PhU5 films. a) IR spectra of hybrid films within amide I and II regions. The dashed lines correspond to the position of the amide I and II bands in the PhU5‐W film. b) Contact angle evolution as a result of the hydrophobization.

Analogous evolution was observed for the PhU5 series. Hydrophobization of hybrid films had also an undeniable impact on the position of the amide I and amide II bands (**Figure** **6**a and Table 1). Compared to PhU5‐W film, PhU5‐MTES film exhibits the smallest upshift of Δ = 12 cm^−1^ for the amide I band. One can see that this band exhibits also a component localized around 1665 cm^−1^. This is evidence of heterogeneity at the molecular level—a fraction of ureido species cannot form HB or forms HB of U—U type. At the same time, the other still may interact with the silanols.

For postgrafted samples PhU5‐TCMS and PhU5‐HMDS, the shift of the amide I band increases to 22 and 27 cm^−1^, respectively, compared with the nongrafted, washed PhU5‐W film. Importantly, we also observe that amide II band downshifts for these two samples (Δ = 5 and Δ = 14 cm^−1^, respectively). Undoubtedly, three ways of functionalization resulted in different organizations of the hybrid interface.

We can correlate these results with contact angle measurements which give us information about the hydrophobicity of the samples (Figure 6b). Only a slight increase in contact angle was observed for the PhU5‐MTES sample, which correlates well with only partial replacement of the amide I band. On the other hand, much higher contact angles are observed for PhU5‐HMDS and PhU5‐TCMS which corresponds to bigger shifts in the amide I band. The level of hydrophobization seems to be very close between these two samples (assuming similar grafting density of methyl and equal density of silanols), while the reported displacement of amide bands was stronger for the PhU5‐HMDS sample. This effect is attributed to the steric hindrance of trimethyl groups grafted at the surface, as they play a dual effect by capping the silanol groups and intercalating between PhU moieties (leading to their separation). This seems to be further confirmed by the strong evolution of the amide II band, which was not observed in other systems.

To promote silanol condensation while minimizing steric hindrance, we also evaluated treatment with NH_3_ vapors. However, the results were inconclusive and are discussed in more detail in Figure S8, Supporting Information.

### 2.4 Deconvolution of the IR Spectra

Based on the discussed differences between spectra and the evidence of distinct molecular environments, we can expect that for each system, the amide I peak is composed of several bands corresponding to these environments. Indeed, DFT calculations (see Table 2 and Figure S9, Supporting Information) show that free carbonyls are upshifted compared to those involved in the formation of a ureido–ureido dimer. On the other hand, carbonyl forming the HB with surfacial silanols should be downshifted.

The experimental amide I band is composed of a larger number of vibrational modes, indicating the high complexity of the molecular environment. Yet, the deconvolution of this signal into all possible components is subject to the risk of error. Moreover, it may make qualitative interpretation of the results difficult, or even impossible. To bypass this ambiguity, we can approximate the amide I band as a sum of the three components corresponding to those found by DFT calculations, bearing in mind that the investigated systems are much more complex.

**Figure 7:**
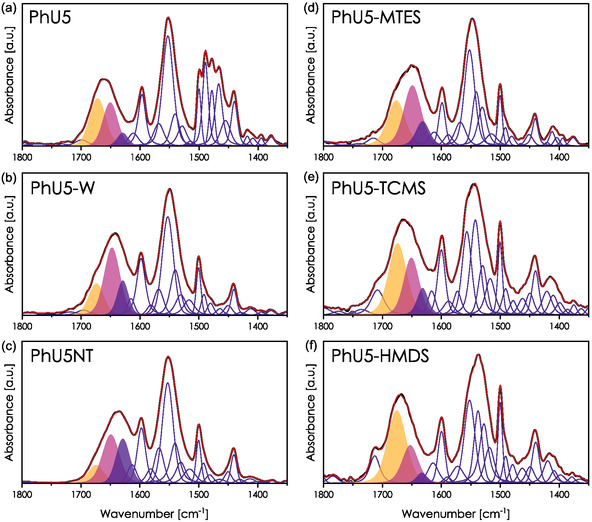
Deconvolution of IR spectra of hybrid films. The three main components of the amide I band are marked to visualize better their evolution: U in yellow, U—U in pink, and SiOH—U in purple.

The IR spectra of PhU5, PhU5‐W, and PhU5NT were deconvoluted into vibrational bands of partial Gaussian and Lorentzian character (**Figure** **7**). For each sample, the amide I band is presented as a sum of three components localized around 1675, 1650, and 1630 cm^−1^, and corresponding to free ureido groups, H‐bonded ureido groups, and ureido groups interacting with surfacial silanols. These bands will be referred to as U, U—U, and SiOH—U. In this regard, the initial shift observed between PhU5 and PhU5‐W samples after the CTAB removal originates from the decrease in the population of free (separated) moieties (U band) and the increase in those forming HBs (U—U and SiOH—U bands). On the other hand, the intensity of the SiOH—U band is substantially higher for the PhU5NT film. The very low intensity of the U band is explained by the low probability of HB cleavage during dip‐coating. When there is no surfactant, molecules are not pushed by any force to separate.

The amide I band can be analyzed in the same way for hydrophobized systems PhU5‐HMDS, PhU5‐TCMS, and PhU5‐MTES. Compared with the PhU5‐W spectrum, we see an increase in the U band for the PhU5‐MTES sample, which results in the mentioned bulky shoulder. The amide I band of the PhU5‐TCMS and PhU5‐HMDS films is dominated by vibrations in free ureido groups. The intensity of the U—U signal for PhU5‐HMDS is visibly reduced, and the SiOH—U band nearly disappears. This effect is associated with possible steric hindrance induced by trimethyl groups. Therefore, the shifts discussed in the previous section should be regarded rather as the change in the relative intensity of several vibrations. The deconvolution also suggests that bonded and nonbonded molecules exist at all stages before the template removal, but their proportion evolves. This again is coherent with the behavior of hybrid films containing pyrene moieties.^[^ ^32^ ^]^ For analogous concentration of pyrene (5%), we observed the simultaneous presence of isolated and interacting pyrene before and after the template removal. The main difference was only the proportion between these two fractions. At lower concentrations (<1%), it was possible to observe only isolated molecules. Therefore, performing a similar study of systems containing different concentrations of PhU‐TES precursor could bring more insight into the organization at the molecular level.

### 2.5 Influence of the Precursor's Respective Concentration

**Figure 8:**
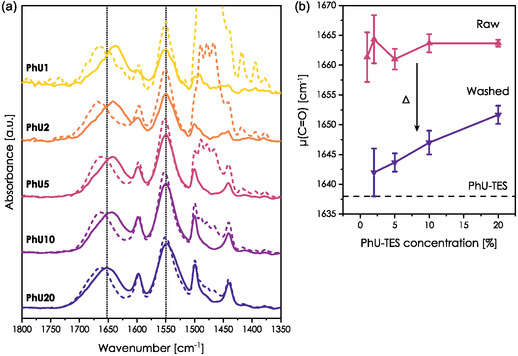
a) IR spectra of hybrid films containing various amounts of PhU‐TES (between 1% and 20%). Dashed and solid lines correspond to as‐prepared (raw) and washed films, respectively. b) Evolution of amide I position as a function of the precursor concentration.

A series of films with increasing PhU‐TES concentration was prepared to verify if one type of bond prevails at a given grafting density. Raw and washed hybrid samples are denoted as PhUX and PhUX‐W, where *X* = 1, 2, 5, 10, 20 corresponds to the PhU‐TES/Si molar ratio. The IR spectra of the amide I and II regions, normalized to the C=O band, are presented in **Figure** **8**a. (full IR spectra normalized to (Si—O—Si) vibration are available in Figure S10, Supporting Information)

Regardless of the PhU‐TES concentration, we observed the downshift of the amide I band after the template removal in all hybrid samples. The initial and final positions of the bands vary. Figure 8 presents the average position of the amide I band (based on several spectra) as a function of the precursor's loading for raw and washed samples. Relatively big error bars for low concentrations originate from possible overlap with the residual signal of (H—O—H) scissoring and high background/signal ratio. The position of the amide I band for unwashed films is independent of the PhU‐TES concentration (about 1664 cm^−1^) but evolves after the template removal. For PhU2 film amide I shifts to 1642 cm^−1^ after washing (Δ = 22 cm^−1^). On the other hand, for the sample with the highest concentration, it downshifts only to 1652 cm^−1^ (Δ = 12 cm^−1^).

From the position of the initial peak, we can conclude that CTAB chains are effective in screening the HB formation within a wide range of concentrations. For the washed films, the *δ*(C=O) vibration upshifts with PhU‐TES concentration moving away from the position of pure PhU‐TES (dashed horizontal line). It may seem counterintuitive, as one would expect the systems of higher PhU‐TES concentration to behave more similarly to a pure precursor. Yet, we must bear in mind the possibility of the formation of SiOH—U bonds, which are characterized by vibrations at a lower wavenumber. The relation between the band position and concentration may suggest that this is the preferential type of bonding in the studied systems. This also explains the biggest value of Δ observed for PhU1 and PhU2 films. Once accessible silanols are saturated, molecules form U—U bonds, and for the highest concentrations, a significant fraction remains in a free state, as they do not have counterparts for HB formation. For high loading, a certain number of molecules may be embedded within the inorganic framework.^[^ ^78^ ^]^

**Figure 9:**
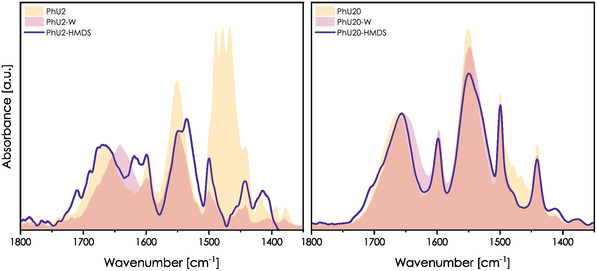
IR spectra of PhU2‐HMDS and PhU20‐HMDS films after the hydrophobization (purple lines). Spectra of raw (yellow) and washed films (pink) are presented in the background for comparison.

To evaluate this hypothesis, we performed posttreatment with HMDS on PhU2 and PhU20 samples. Spectra of PhU2‐HMDS and PhU20‐HMDS films are presented in **Figure** **9**. The spectra of the PhU2‐HMDS and PhU2 samples overlap almost ideally within the amide I region. In the case of the Phu20‐HMDS sample, we state a slight shift for amide I (between PhU20 and PhU20‐W spectra) coupled with the band stretching toward a higher wavenumber. It supports the hypothesis that low precursor proportion favors the formation of SiOH—U bonds, while high loading leads to U—U dimers and free U moieties.

We see here that exploring the IR spectra of films with precursor proportions lower than 2% is limited, as the intensity of amide I and II bands becomes too weak. This is an unfortunate obstacle, as the most interesting phenomena for pyrene‐containing films were reported for loading of about 1%. It is for low concentrations that we can observe a definite predominance of one type of organization (e.g, dimer vs free molecule). While IR spectroscopy imposes this concentration limit, fluorescence spectroscopy provides excellent insight into the structure of hybrid materials with low grafting density. Hence, a synthesis of dual‐functionality probes that could be investigated by both spectroscopies would be extremely profitable. Systems based on such molecules will be discussed in a forthcoming article.

A series of samples with increasing concentrations of hybrid precursor give us also an opportunity to perform a comparative study of structural properties (which can be easily accessed through ellipsoporosimetry).^[^ ^79^ ^]^ It was shown that introducing hybrid species (even of simple geometry, such as alkyl) substantially alters the mesophase type, pore size, micro‐ and mesopore volume, or Young's modulus. In past works, we and others have evidenced that hybrid silane molecules may act as cotemplating agents, altering the micelle curvature (and therefore the pore geometry and pore arrangement).^[^ ^78^ ^]^ At low concentrations, silane species are placed preferably within the micelle core (if it is allowed by the molecule's size and polarity), but at higher loading, they also populate the hybrid interface and the inorganic framework, which is observed as a decrease of Young's modulus of mesostructured materials.^[^ ^78^ ^]^

**Figure 10:**
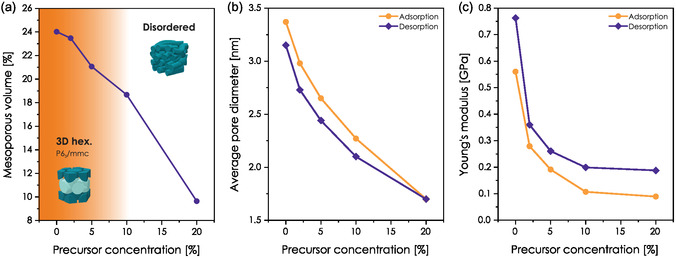
Influence of Phu‐TES concentration on the film's mesostructure. a) Mesoporous volume and mesostructure evolution. b) Average pore size, and c) Young's modulus determined from ellipsometry. Lines connecting the points are added to better visualize the trends.

**Figure** **10** presents how the mesophase, pore size, mesoporous volume, and Young's modulus evolve with the concentration of PhU‐TES in washed hybrid films. A dedicated grazing‐incidence small‐angle scattering (GI‐SAXS) study of our materials evidenced that the initial 3D hexagonal (P6_3_/mmc) arrangement of mesopores is maintained for the films with PhU concentration up to 5%, and a wormlike structure is obtained for higher loadings (see Figure S11, Supporting Information). Conserving the 3D hexagonal arrangement may be possible due to the partially hydrophobic character of the PhU moiety induced by the presence of the terminal phenyl ring. This could promote its intercalation in the deeper, hydrophobic regions of the micelle, without disturbing the curvature of the hybrid interface.^[^ ^31^ ^]^

The average mesopore size decreases with increasing hybrid precursor concentration, from a reference value of 3.37 nm in the nonhybrid system to 1.7 nm in the PhU20 sample (Figure 10b). This reduction is particularly pronounced at low precursor concentrations, a trend commonly observed in hybrid mesoporous materials.^[^ ^78^ ^]^ At the same time, we observe a decrease in the mesoporous volume, but the drop in this value is less abrupt for smaller concentrations. It can be attributed to the co‐templating effect of PhU moiety. For a certain range of concentrations, the decrease of mesoporosity caused by the presence of hybrid species may be balanced by an increase in the number of pores (micelles) induced by the cotemplating effect of the precursor.^[^ ^78^ ^]^

On the other hand, we observe a decrease in Young's modulus, which is typically associated with the entrapment of organic functions within the inorganic framework. Films with high concentrations of hybrid precursor are particularly susceptible to it, as the available sites within the pore interface become saturated, and organic species may be forced to be embedded in the pore wall. The increase in framework flexibility is also suspected to come from the overall lower level of network condensation, as fewer Si—O—Si bridges can be formed due to the presence of Si—R bonds.

### 2.6 Study of the Phenylureido Ternary Systems

**Figure 11:**
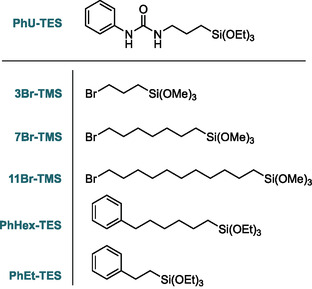
Structure of trialkoxysilane precursors used in ternary systems and abbreviations of their names: 3‐bromopropyltrimethoxysilane (3Br‐TMS), 7‐bromoheptyltrimethoxysilane (7Br‐TMS), 11‐bromoundecyltrimethoxysilane (11Br‐TMS), [6‐(triethoxysilyl)hexyl]benzene (PhHex‐TES), and (2‐phenylethyl)trimethoxysilane (PhEt‐TES).

In ternary systems (composed of a nonhybrid and two distinct hybrid precursors) also functional groups of different chemical natures can still interact within the mesopore. We reported that the formation of pyrene excimers could be successfully hindered by introducing a ternary moiety (e.g., alkyl chain) simultaneously during the co‐condensation.^[^ ^32^ ^]^ Following this concept, we prepared several samples using TEOS as a primary precursor, PhU‐TES as a secondary precursor, and an additional hybrid molecule as a ternary precursor. In the first series, 5% of PhU‐TES was mixed with 10% of bromoalkyl precursors with different chain lengths to generate a mild steric hindrance that could reduce the level of H‐bonding. Moreover, to induce stronger interactions between two hybrid precursors, we prepared films with ternary precursors of aromatic character. These films contain 5 mol% of PhU‐TES coupled with (2‐phenylethyl)trimethoxy‐silane, or [6‐(triethoxysilyl)hexyl]benzene. Structures of all hybrid precursors and their abbreviations are presented in **Figure** **11**, and the exact composition of the synthesized films is listed in **Table** **3**.

**Table 3:** Composition of systems based on tetralkoxysilane, PhU‐TES, and a ternary precursor.

Film	PhU‐TES [%]	Ternary precursor	Concentration [%]
PhU5‐3Br10‐W	5	3Br‐TMS	10
PhU5‐7Br10‐W	5	7Br‐TMS	10
PhU5‐11Br10‐W	5	11Br‐TMS	10
PhU5‐PhEt5‐W	5	EtPh‐TES	5
PhU5‐PhHex5‐W	5	HexPh‐TES	5
PhU2‐7Br10‐W	2	11Br‐TMS	10

**Figure 12:**
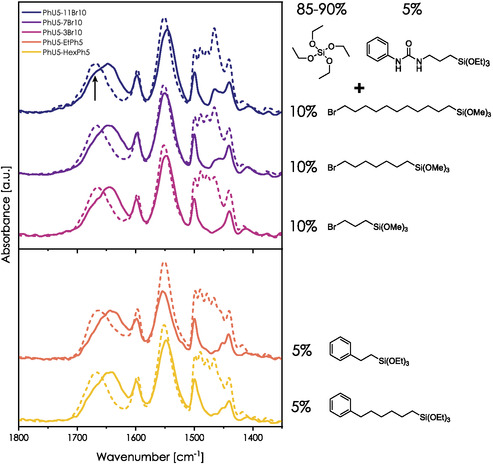
Spectra of raw (dashed lines) and washed (solid lines) films composed of one nonhybrid and two hybrid precursors. Top: Combination of 5 mol% of PhU‐TES and 10 mol% of bromoalkyl precursors; bottom: 5 mol% of PhU‐TES and 5 mol% of aromatic precursors. Dashed and solid lines correspond to as‐prepared (raw) and washed films, respectively.

**Figure** **12** presents the FTIR spectra of raw and extracted ternary films. The shift of amide I induced by the template elimination is present regardless of the bromoalkyl chain length and precursor loading. While the peak position of the amide I band does not vary significantly for PhU5‐3Br10‐W, PhU5‐7Br10‐W, and PhU5‐11Br10‐W, some evolution can be stated. We observe an increase in intensity for a peak component localized around 1670 cm^−1^ compared to the PhU5‐W sample. This band (marked with an arrow) corresponds to carbonyl vibrations in free ureido species. A similar effect was observed for the PhU5‐MTES sample, which can also be considered as a “ternary system.” Here, this band is the most pronounced for PhU5‐11Br10‐W and less visible in the case of PhU5‐3Br10‐W. Obviously, the chain length seems to be an important parameter in restricting interactions between the ureido groups.

The following two systems, PhU5‐PhEt5‐W and PhU5‐PhHex5‐W, aimed to verify if HB formation can be restricted more by using a ternary precursor with potential additional interactions with the PhU group (induced by the π–π stacking between aromatic rings). Indeed, no important reduction of amide I shift is observed for these systems (Figure 12, bottom panel). This is quite unexpected, considering the affinity between the precursors.

For PhU5‐PhEt5‐W film, the lack of the desired effect may be explained by two big differences in the lengths of Si‐Ph linkers. For PhEt‐TES, the ethyl chain between benzene and the silicon atom may be too short to allow π–π stacking with the aromatic ring of the PhU. On the other hand, the spacer of the PhHex‐TES precursor has a length comparable to that of PhU‐TES. In this case, the lack of an impact on HB formation may be attributed to the flexibility of hexyl chains, which lowers the probability of achieving stable π–π interaction for entropic reasons. We can also consider a segregation effect between PhU and PhHex/PhEt moieties within the pores. The separation of two precursors into two different regions of a single cavity would be a remarkable phenomenon. Unfortunately, experimental verification of this hypothesis is not trivial.

The modest effectiveness of ternary precursors in blocking the HB formation may also be explained by a small difference in concentration of two hybrid precursors (ratio 1:2 and 1:1). For instance, in our past work we observed strong excimer hindrance for films containing 5 mol% of pyrene when the ternary precursor was introduced in 3 times higher loading.^[^ ^32^ ^]^ Yet, it was still not sufficient to block the π–π stacking completely.

**Figure 13:**
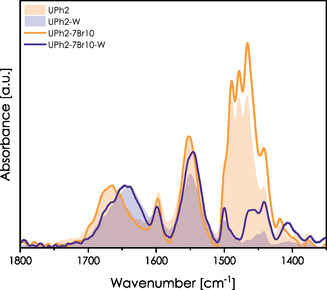
Spectra of PhU2‐7Br10 and PhU2‐7Br10‐W samples. For reference, PhU2 and PhU2‐W spectra are presented in the background. Spectra are normalized to the amide I band.

Finally, we prepared a system containing 2 mol% of PhU‐TES and 10 mol% of 7Br‐TMS. **Figure** **13** compares spectra of PhU2‐7Br10 and PhU2‐7Br10‐W films with their binary counterparts (PhU2). For raw films, the amide I and II bands overlap almost ideally. In the case of washed films, only a slight increase of amide II and a minimal upshift of amide I band are observed for the ternary system. Therefore, the presence of the bromoalkyl chain seems not to disturb the HB formation, which may be caused by molecular segregation within the pores. On the other hand, as already discussed, low precursor concentration may privilege the SiOH—U bonds, which would not be affected by the ternary precursor. Such a bond is formed directly at the interface, and its hindrance by the alkyl chain is improbable.

## 3 Conclusion

In this work, we presented the investigation of the molecular interactions in confined environments based on HB formation. We described the phenylureido precursor and the way it can interact in silica pores. We observed HB formation after the extraction of the template from PhU films. The formation of ureido HBs was observed through IR spectroscopy as an upshift of the amide I band and a downshift of the amide II bands.

In mesostructured materials, the HB formation is restricted by the presence of surfactants. Once it is eliminated, HB may be formed as evidenced by the observation of an upshift of the amide I band. This effect is analogous to pyrene's excimer formation discussed in our previous work, indicating the existence of a general law governing the hybrid self‐assembly. A particular feature of the studied systems is the possibility of interactions within the interface as ureido‐silanol bonds. We proposed several methods to differentiate whether bonds form between two ureido groups or between an ureido group and a silanol moiety. Using these approaches, we successfully deconvoluted the amide I band into three distinct components: free ureido groups, ureido–ureido dimers, and ureido groups involved in hydrogen bonding with silanols. The overall shifts observed in the amide I region are due to changes in the relative intensity of these components over time.

The formation of HBs was studied in samples with different concentrations of the hybrid precursor. While for all films we reported the evolution of the amide I band upon the template removal, the scale of the observed shift depends on the precursor's proportion. High loading promotes the formation of U—U bonds and the existence of free ureido species, while at low concentrations, the SiOH—U bond seems to prevail. The molecular structure of the precursor itself, and in particular the presence of a terminal aromatic group, is also of key importance. The exact role and impact of these parameters on the architecture of hybrid films will be discussed in detail in our forthcoming work.

In ternary systems, we observed a partial hindrance of HB formation due to the presence of bromoalkyl moieties. However, a complete blockage of interactions was not possible, which may suggest the segregation of two molecular probes within the pore.

Investigating the molecular environment in hybrid mesoporous films is a challenging but crucial task. Weak interactions between hybrid species may hold the key to understanding these systems. While HB formation involving ureido species provides some insights, secondary effects, such as ureido–silanol interactions, must also be considered. The limitations of IR spectroscopy hinder the study of systems with very low precursor loading. However, this will be addressed in the future by developing dual‐functionality probes that combine other moieties with ureido species in a single hybrid precursor, opening new possibilities for investigation.

## 4 Experimental Section

4.1

4.1.1

Tetraethoxysilane was purchased from ThermoFisher; methyltriethoxysilane, (2‐phenylethyl)trimethoxysilane and 3‐bromopropyltrimethoxysilane from Abcr; 7‐bromoheptyltrimethoxysilane and 11‐bromoundecyltrimethoxysilane from Gelest; (3‐isocyanatopropyl)triethoxysilane, cetyltrimethylammonium bromide (CTAB), triethoxysilane, aniline, trichloromethylsilane, and Karstedt's catalyst (in xylene, Pt ≈ 2%) from Sigma–Aldrich; and hexamethyldisilazane from Alfa Aesar. All chemicals were utilized without any supplementary purification.

##### Synthesis of the Organosilane Precursor N‐Phenyl‐N′‐[3‐(triethoxysilyl)propyl]urea

The compound was prepared in the same conditions according to the protocol published by Pichon et al.^[^ ^80^ ^]^ Under an argon atmosphere, 3‐isocyanatopropyltriethoxysilane (2 mmol) was added to a solution of aniline (2 mmol) in dry THF (4 mL). An exothermic effect was observed while mixing the reagents. The mixture was heated for two hours at 60 °C. The solvent was removed under vacuum, and the crude product was taken in pentane (5 mL). After the filtration and crystallization, products were obtained quantitatively. ^1^HNMR (300 MHz, CDCl_3_) *δ* = 0.63 (t, *J* = 8.1 Hz, 2H), 1.19 (t, *J* = 7.0 Hz, 9H), 1.63 (m, 2H), 3.24 (q, *J* = 6.6 Hz, 2H), 3.79 (q, *J* = 7.0 Hz, 6H), 4.18 (s, 1H), 6.15 (s, 1H), 7.04‐7.34 (m, 5H). Full ^1^H NMR spectra are available in the SI.

##### Synthesis of the Organosilane Precursor [6‐(Triethoxysilyl)hexyl]benzene

Triethoxysilane (0.43 g, 2.6 mmol) and 6‐phenyl‐1‐hexene (0.625 g, 3.9 mmol) were added to dry toluene (20 mL) under an argon atmosphere. Karstedt's catalyst (0.01 mL) was added dropwise, and the solution was heated overnight at 70 °C. Toluene was removed under a vacuum, and the filtration on silica eluting with DCM gave pure [6‐(triethoxysilyl)hexyl]benzene as a white solid (0.79 g, yield: 91%). ^1^H NMR (300 MHz, CDCl_3_) *δ* = 0.61 (t, *J* = 7.9 Hz, 2H), 1.21 (t, *J* = 7.0 Hz, 9H), 1.36 (m, 6H), 1.60 (m, 2H), 2.58 (t, *J* = 7.7 Hz, 2H), 3.79 (q, *J* = 7.7 Hz, 6H), 7.20 (m, 5H). Full ^1^H NMR spectra are available in the SI.

##### Preparation of Silica Films

Thin mesoporous silica films were prepared using the EISA method. Initial sols were synthesized via co‐condensation, following the protocol established in our group.^[^ ^31^, ^81^ ^]^

All silica precursors, ethanol, water, and hydrochloric acid, were stirred for 1 h at room temperature (molar ratios 1–*x* TEOS:*x* PhU‐TES:3 EtOH:1 H_2_O:5.10^−5^ HCl). Subsequently, a second solution containing the templating agent, ethanol, and an aqueous solution of HCl was added, resulting in final molar ratios of 1–*x* TEOS:*x* PhU‐TES:0.1 CTAB:20 EtOH:5 H_2_O:0.004 HCl. The maximum value of *x* is limited by both porosity and mesostructural order. Depending on the chosen precursor, porosity can be lost at *x* = 0.2, while the structural organization is hindered at even lower concentrations (*x* ≈ 0.1).^[^ ^78^ ^]^ Consequently, for systems containing PhU‐TES, we used a maximum *x* value of 0.05. Throughout the article, the concentration of PhU‐TES refers to the molar ratio between PhU‐TES and whole silica source

(1) $$\frac{\text{PhU‐TES}}{\text{PhU‐TES+TEOS}}$$

The solutions were aged at RT for 2 days before the fabrication of the coatings. When stored at 5 °C between usages, they remain suitable for film preparation up to one month after synthesis. Thin films were fabricated using the dip‐coating technique in a closed chamber allowing the precise control of relative humidity (RH), which is a crucial factor determining the final mesostructure.^[^ ^31^ ^]^ During the coating and the three following minutes, RH was fixed at 40 ± 2%. The temperature inside the chamber was kept at 22 ± 1 °C. Withdrawal speed was set to 8 mm s^−1^ to ensure sufficient thickness and high optical quality of the coating. Films were stabilized thermally at 130 °C for 3 days, followed by template extraction, which was achieved by immersing the samples in a flask containing 30 mL of absolute ethanol for 15 min and then rinsing afterward.

##### DFT Quantum Chemical Calculations

The Gaussian16 code^[^ ^82^ ^]^ was used to optimize molecular geometries along with the global hybrid functional PBE0.^[^ ^83^ ^]^ Structural optimizations and subsequent frequency calculations were performed using an all‐electron Pople triple zeta basis set with one polarization function on all atoms and one diffuse function of heavier atoms, known as 6–311+G(d,p), for H, C, N, and O atoms. Bulk solvent effects were included using the polarizable continuum model of Tomasi and co‐workers. Default radii (from the UFF, scaled by 1.1) were used. Vibrational frequencies were then scaled by a 0.959 factor.^[^ ^84^ ^]^

##### Analysis

IR spectra were recorded using a PerkinElmer Spectrum 65 FT‐IR spectrometer equipped with an ATR attachment with a single‐reflection diamond crystal. The hybrid films were scratched with a scalpel, and the obtained powder was put directly in contact with the ATR prism surface. Data were collected with 4 scans from 4000 to 600 cm^−1^.

The Wollam M‐2000 ellipsometer was used for ellipsometric measurements. Data fitting was performed using the CompleteEASE software, employing a multilayer model with a fixed substrate consisting of Si, a 10 Å interlayer, a 20 Å SiO_2_ layer, and a 750 Å Ti/Cr layer. The properties of the thin film (top layer) were modeled using the Cauchy model, with the following initial parameters: *A* = 1.3, *B* = 0.003, and thickness *h* = 4000Ǻ. Additionally, the fit was corrected for film thickness nonuniformity. The derived parameters, film thickness (*h*) and refractive index (*n*), were calculated at a wavelength of 700 nm.

For ellipsoporosimetry, we employed pure isopropanol vapors as adsorbents to ensure good affinity to hydrophobic pores. Isotherms were extracted from the evolution of film refractive index *n* at 700 nm wavelength. The relative pressure of isopropanol in the chamber was raised from 0% to 60% over 15 min and brought down at the same rate. Detailed methodology of mesostructure analysis through ellipsoporosimetry is provided elsewhere.^[^ ^32^, ^78^, ^79^ ^]^

GI‐SAXS measurements were done with Xesuss beamline (Xenocs). Measurements of the contact angle were performed with a Drop Shape Analyzer (Krüss) using distilled water as probe liquid.

## Conflict of Interest

The authors declare no conflict of interest.

## Supporting information

Supplementary Material

## Data Availability

The data that support the findings of this study are available from the corresponding author upon reasonable request.

## References

[1] H. J. Gardeniers , Anal. Bioanal. Chem. 2009, 394, 385. 19234693 10.1007/s00216-009-2672-5

[2] A. B. Grommet , M. Feller , R. Klajn , Nat. Nanotechnol. 2020, 15, 256. 32303705 10.1038/s41565-020-0652-2

[3] J. L. Defreese , S. J. Hwang , A. N. Parra‐Vasquez , A. Katz , J. Am. Chem. Soc. 2006, 128, 5687. 16637635 10.1021/ja0556474

[4] Y. Wu , G. Cheng , K. Katsov , S. W. Sides , J. Wang , J. Tang , G. H. Fredrickson , M. Moskovits , G. D. Stucky , Nat. Mater. 2004, 3, 816. 15502836 10.1038/nmat1230

[5] J. Jiou , K. Chiravuri , A. Gudapati , J. Gassensmith , Curr. Org. Chem. 2014, 18, 2002.

[6] G. Tabacchi , ChemPhysChem 2018, 19, 1249. 29573368 10.1002/cphc.201701090

[7] Y. Ren , Z. Zhou , K. Maxeiner , A. Kaltbeitzel , I. Harley , J. Xing , Y. Wu , M. Wagner , K. Landfester , I. Lieberwirth , T. Weil , D. Y. W. Ng , J. Am. Chem. Soc. 2024, 146, 11991. 38639465 10.1021/jacs.4c01279 PMC11066860

[8] M. Wang , A. Wang , J. Li , Q. Li , S. Bai , Colloids Surf. A 2020, 603, 125213.

[9] A. Méndez‐Ardoy , J. R. Granja , J. Montenegro , Nanoscale Horiz. 2018, 3, 391. 32254126 10.1039/c8nh00009c

[10] Q. Dong , M. Wang , A. Wang , C. Yu , S. Bai , J. Yin , Q. You , Biomater. Sci. 2022, 10, 1470. 35170621 10.1039/d2bm00041e

[11] B. Qin , J. F. Xu , X. Zhang , Langmuir 2022, 38, 4157. 35344363 10.1021/acs.langmuir.2c00065

[12] A. G. Slater , L. M. Perdigão , P. H. Beton , N. R. Champness , Acc. Chem. Res. 2014, 47, 3417. 25330179 10.1021/ar5001378

[13] D. Bonifazi , S. Mohnani , A. Llanes‐Pallas , Chemistry 2009, 15, 7004. 19569139 10.1002/chem.200900900

[14] K. Ariga , A. Vinu , J. P. Hill , T. Mori , Coor. Chem. Rev. 2007, 251, 2562.

[15] L. B. Sun , J. R. Li , W. Lu , Z. Y. Gu , Z. Luo , H. C. Zhou , J. Am. Chem. Soc. 2012, 134, 15923. 22937898 10.1021/ja3063925

[16] L. J. Chen , S. Chen , Y. Qin , L. Xu , G. Q. Yin , J. L. Zhu , F. F. Zhu , W. Zheng , X. Li , H. B. Yang , J. Am. Chem. Soc. 2018, 140, 5049. 29625011 10.1021/jacs.8b02386

[17] Y.‐H. Kang , X.‐D. Liu , N. Yan , Y. Jiang , X.‐Q. Liu , L.‐B. Sun , J.‐R. Li , J. Am. Chem. Soc. 2016, 138, 6099. 27049737 10.1021/jacs.6b01207

[18] P. Picchetti , G. Moreno‐Alcantar , L. Talamini , A. Mourgout , A. Aliprandi , L. De Cola , J. Am. Chem. Soc. 2021, 143, 7681. 33891394 10.1021/jacs.1c00444

[19] E. D. Licsandru , S. Schneider , S. Tingry , T. Ellis , E. Moulin , M. Maaloum , J. M. Lehn , M. Barboiu , N. Giuseppone , Nanoscale 2016, 8, 5605. 26892311 10.1039/c5nr06977g

[20] K. Sentker , A. Yildirim , M. Lippmann , A. W. Zantop , F. Bertram , T. Hofmann , O. H. Seeck , A. V. Kityk , M. G. Mazza , A. Schonhals , P. Huber , Nanoscale 2019, 11, 23304. 31788679 10.1039/c9nr07143a

[21] G. J. Soler‐Illia , O. Azzaroni , Chem. Soc. Rev. 2011, 40, 1107. 21221447 10.1039/c0cs00208a

[22] A. Andrieu‐Brunsen , S. Micoureau , M. Tagliazucchi , I. Szleifer , O. Azzaroni , G. J. A. A. Soler‐Illia , Chem. Mater. 2015, 27, 808.

[23] S. Alberti , G. J. Soler‐Illia , O. Azzaroni , Chem. Commun. 2015, 51, 6050. 10.1039/c4cc10414e 25675435

[24] A. Brunsen , C. Diaz , L. I. Pietrasanta , B. Yameen , M. Ceolin , G. J. Soler‐Illia , O. Azzaroni , Langmuir 2012, 28, 3583. 22309103 10.1021/la204854r

[25] T. D. Nguyen , K. C.‐F. Leung , M. Liong , C. D. Pentecost , J. F. Stoddart , J. I. Zink , Org. Lett. 2006, 8, 3363. 16836406 10.1021/ol0612509

[26] K. C.‐F. Leung , T. D. Nguyen , J. F. Stoddart , J. I. Zink , Chem. Mater. 2006, 18, 5919.

[27] Y. Wu , K. Luo , Y. Liu , W. Chen , Z. Bai , S. Tang , J. Chromatogr. A 2022, 1665, 462834. 35085896 10.1016/j.chroma.2022.462834

[28] S. Huh , H. T. Chen , J. W. Wiench , M. Pruski , V. S. Lin , Angew. Chem. Int. Ed. 2005, 44, 1826. 10.1002/anie.200462424 15712306

[29] A. Calvo , M. Joselevich , G. J. A. A. Soler‐Illia , F. J. Williams , Microporous Mesoporous Mater. 2009, 121, 67.

[30] A. Darga , J. Kecht , T. Bein , Langmuir 2007, 23, 12915. 18001067 10.1021/la701962b

[31] D. Grosso , F. Cagnol , G. J. D. A. A. Soler‐Illia , E. L. Crepaldi , H. Amenitsch , A. Brunet‐Bruneau , A. Bourgeois , C. Sanchez , Adv. Funct. Mater. 2004, 14, 309.

[32] J. Kusz , C. Boissiere , Y. Bretonnière , C. Sanchez , S. Parola , Nanoscale 2024, 16, 18918. 39267607 10.1039/d4nr02987a

[33] G. R. Desiraju , Angew. Chem. Int. Ed. 2011, 50, 52. 10.1002/anie.201002960 21031379

[34] F. Lortie , S. Boileau , L. Bouteiller , Chemistry 2003, 9, 3008. 12833282 10.1002/chem.200304801

[35] R. Custelcean , Chem. Commun. 2008, 3, 295. 10.1039/b708921j 18401890

[36] M. M. Nieuwenhuizen , T. F. de Greef , R. L. van der Bruggen , J. M. Paulusse , W. P. Appel , M. M. Smulders , R. P. Sijbesma , E. W. Meijer , Chemistry 2010, 16, 1601. 20039341 10.1002/chem.200902107

[37] M. Obrzud , M. Rospenk , A. Koll , Phys. Chem. Chem. Phys. 2014, 16, 3209. 24406348 10.1039/c3cp53582g

[38] M. A. Ramin , G. Le Bourdon , K. Heuze , M. Degueil , C. Belin , T. Buffeteau , B. Bennetau , L. Vellutini , Langmuir 2012, 28, 17672. 23189954 10.1021/la303805d

[39] M. A. Ramin , G. Le Bourdon , K. Heuze , M. Degueil , T. Buffeteau , B. Bennetau , L. Vellutini , Langmuir 2015, 31, 2783. 25679263 10.1021/la5049375

[40] M. Meillan , T. Buffeteau , G. Le Bourdon , L. Thomas , M. Degueil , K. Heuzé , B. Bennetau , L. Vellutini , Chem. Select 2017, 2, 11868.

[41] J. J. Moreau , L. Vellutini , M. Wong Chi Man , C. Bied , J. L. Bantignies , P. Dieudonne , J. L. Sauvajol , J. Am. Chem. Soc. 2001, 123, 7957. 11493090 10.1021/ja016053d

[42] J. J. Moreau , B. P. Pichon , M. Wong Chi Man , C. Bied , H. Pritzkow , J. L. Bantignies , P. Dieudonne , J. L. Sauvajol , Angew. Chem. Int. Ed. 2004, 43, 203. 10.1002/anie.200352485 14695609

[43] J. L. Bantignies , L. Vellutini , J. L. Sauvajol , D. Maurin , M. Wong Chi Man , P. Dieudonné , J. J. E. Moreau , J. Non‐Cryst. Solids 2004, 345–346, 605.

[44] R. Le Parc , V. T. Freitas , P. Hermet , A. M. Cojocariu , X. Cattoen , H. Wadepohl , D. Maurin , C. H. Tse , J. R. Bartlett , R. A. S. Ferreira , L. D. Carlos , M. Wong Chi Man , J. L. Bantignies , Phys. Chem. Chem. Phys. 2019, 21, 3310. 30688324 10.1039/c8cp06625f

[45] M. Benitez , D. Das , R. Ferreira , U. Pischel , H. García , Chem. Mater. 2006, 18, 5597.

[46] J. He , Z. Liu , C. Hai , AIChE J. 2008, 54, 2495.

[47] W. Bicker , J. Wu , H. Yeman , K. Albert , W. Lindner , J. Chromatogr A 2011, 1218, 882. 21067765 10.1016/j.chroma.2010.10.073

[48] M. Moritz , M. Geszke‐Moritz , Mater. Sci. Eng. C: Mater. Biol. Appl. 2014, 41, 42. 24907735 10.1016/j.msec.2014.04.032

[49] D. Lewandowski , D. Bajerlein , G. Schroeder , Struct. Chem. 2014, 25, 1505.

[50] A. S. Timin , E. V. Rumyantsev , A. V. Solomonov , I. I. Musabirov , S. N. Sergeev , S. P. Ivanov , G. Berlier , E. Balantseva , Colloids Surf., A 2015, 464, 65.

[51] M. Zhou , W. Tang , P. Luo , J. Lyu , A. Chen , L. Qiao , D. Nover , Water Sci. Technol 2017, 76, 2526. 29144310 10.2166/wst.2017.405

[52] S. Bao , Y. Li , Z. Fei , H. Mei , Y. Zhou , X. Wang , D. Liu , J. Radioanal. Nucl. Chem. 2020, 324, 385.

[53] V. Amendola , L. Fabbrizzi , L. Mosca , Chem. Soc. Rev. 2010, 39, 3889. 20818452 10.1039/b822552b

[54] J. R. Hiscock , N. J. Wells , J. A. Ede , P. A. Gale , M. R. Sambrook , Org. Biomol. Chem. 2016, 14, 9560. 27722624 10.1039/c6ob01210h

[55] Y. Cao , Y. Fan , Z. Ma , Z. Cheng , Q. Xiang , Z. Duan , J. Xu , Sens. Actuators B: Chem. 2018, 273, 1162.

[56] M. Michau , R. Caraballo , C. Arnal‐Hérault , M. Barboiu , J. Membr. Sci. 2008, 321, 22.

[57] G. Creff , B. P. Pichon , C. Blanc , D. Maurin , J. L. Sauvajol , C. Carcel , J. J. Moreau , P. Roy , J. R. Bartlett , M. Wong Chi Man , J. L. Bantignies , Langmuir 2013, 29, 5581. 23574041 10.1021/la400293k

[58] Y. Sun , Z. Sun , C. Wang , Y. Wei , J. Chromatogr A 2022, 1674, 463152. 35597197 10.1016/j.chroma.2022.463152

[59] J. Sano , S. Habaue , Polymers 2020, 12, 1175. 32443903 10.3390/polym12051175 PMC7284717

[60] S. Cerneaux , N. Hovnanian , J. Membr. Sci. 2005, 247, 87.

[61] Y. JunGong , Z. Li , W. U. Dong , Y. Sun , B. Dong , F. Deng , Nanotechnology in Mesostructured Materials of Studies in Surface Science and Catalysis, (Eds: S.‐E. Park , R. Ryoo , W.‐S. Ahn , C. W. Lee , J.‐S. Chang ), Vol. 146, Elsevier, Amsterdam, New York 2003, pp. 461–464.

[62] H.‐T. Chen , B. G. Trewyn , J. W. Wiench , M. Pruski , V. S. Y. Lin , Top. Catal. 2009, 53, 187.

[63] Physical Methods to Characterize Pharmaceutical Proteins, (Eds: E. A. Cooper , K. Knutson , In J. N. Herron , W. Jiskoot , D. J. A. Crommelin ), Springer US, Boston, MA 1995, pp. 101–143.

[64] P. I. Haris , Pharm. Pharmacol. Commun. 1999, 5, 15.

[65] C. Vigano , M. Smeyers , V. Raussens , F. Scheirlinckx , J. M. Ruysschaert , E. Goormaghtigh , Biopolymers 2004, 74, 19. 15137087 10.1002/bip.20035

[66] M. Baldassarre , A. Scirè , F. Tanfani , Biomed. Spectrosc. Imaging 2012, 1, 247.

[67] G. Creff , Thesis, Université Montpellier II 2012.

[68] T. I. Mizan , P. E. Savage , R. M. Ziff , J. Phys. Chem. 1996, 100, 403.

[69] P. Innocenzi , P. Falcaro , D. Grosso , F. Babonneau , J. Phys. Chem. B 2003, 107, 4711.

[70] A. Fidalgo , L. M. Ilharco , Chemistry 2004, 10, 392. 14735508 10.1002/chem.200305079

[71] R. B. Viana , A. B. F. da Silva , A. S. Pimentel , Adv. Phys. Chem. 2012, 2012, 1.

[72] A. Calvo , P. C. Angelomé , V. M. Sánchez , D. A. Scherlis , F. J. Williams , G. J. A. A. Soler‐Illia , Chem. Mater. 2008, 20, 4661.

[73] M. Ide , M. El‐Roz , E. De Canck , A. Vicente , T. Planckaert , T. Bogaerts , I. Van Driessche , F. Lynen , V. Van Speybroeck , F. Thybault‐Starzyk , P. Van Der Voort , Phys. Chem. Chem. Phys. 2013, 15, 642. 23187618 10.1039/c2cp42811c

[74] S. R. Marcsisin , J. R. Engen , Anal. Bioanal. Chem. 2010, 397, 967. 20195578 10.1007/s00216-010-3556-4 PMC2868954

[75] M. Boudot , V. Gaud , M. Louarn , M. Selmane , D. Grosso , Chem. Mater. 2014, 26, 1822.

[76] J. Y. Chen , F. M. Pan , L. Chang , A. T. Cho , K. J. Chao , J. Vac. Sci. Technol., B 2005, 23, 2034.

[77] T. J. Ha , H. H. Park , S. B. Jung , H. Ryu , B. G. Yu , J. Colloid Interface Sci. 2008, 326, 186. 18684463 10.1016/j.jcis.2008.07.024

[78] J. Kusz , C. Boissiere , D. Ihiawakrim , O. Ersen , C. Sanchez , S. Parola , Chem. Mater. 2023, 35, 7671.

[79] C. Boissiere , D. Grosso , S. Lepoutre , L. Nicole , A. B. Bruneau , C. Sanchez , Langmuir 2005, 21, 12362. 16343015 10.1021/la050981z

[80] B. P. Pichon , M. Wong Chi Man , C. Bied , J. J. E. Moreau , J. Organomet. Chem. 2006, 691, 1126.

[81] F. Cagnol , D. Grosso , J. Mater. Chem. 2003, 13, 61.

[82] M. J. Frisch , G. W. Trucks , H. Schlegel , G. E. Scuseria , M. A. Robb , J. R. Cheeseman , G. Scalmani , V. Barone , G. A. Petersson , H. Nakatsuji , X. Li , M. Caricato , A. V. Marenich , J. Bloino , B. G. Janesko , R. Gomperts , B. Mennucci , H. P. Hratchian , J. V. Ortiz , A. F. Izmaylov , J. L. Sonnenberg , D. Williams‐Young , F. Ding , F. Lipparini , F. Egidi , J. Goings , B. Peng , A. Petrone , T. Henderson , D. Ranasinghe , et al., Gaussian 16, Revision C. 01, Gaussian, Inc., Wallingford CT. 2016

[83] C. Adamo , V. Barone , J. Chem. Phys. 1999, 110, 6158.

[84] Y. Tantirungrotechai , K. Phanasant , S. Roddecha , P. Surawatanawong , V. Sutthikhum , J. Limtrakul , J. Mol. Struct.: THEOCHEM. 2006, 760, 189.

