# Supplementary material for: Investigating the Supramolecular Assemblies under the Confinement in Hybrid Mesoporous Silica Films
Source: Small Sci. 2025 Aug 17;5(11):2500369. doi: 10.1002/smsc.202500369 (PMC12622414; doi:10.1002/smsc.202500369)
Supplement: Supplementary file 1 — Supplementary Material [file SMSC-5-2500369-s001.pdf]

Investigating the Supramolecular Assemblies Under the Confinement in Hybrid Mesoporous Silica Films

Jakub Kusz Cédric Boissiere Michel Wong Chi Man*
*Tangui Le Bahers Clément Sanchez Stephane Parola**

Stephane Parola, Tangui Le Bahers
École Normale Supérieure de Lyon, CNRS, Université Claude Bernard Lyon 1, Laboratoire de Chimie, UMR 5182, 46 Allée d'Italie, 69364 Lyon, France
Email Address: stephane.parola@ens-lyon.fr
Cédric Boissiere, Clément Sanchez
Sorbonne Université, CNRS, Collège de France, Laboratoire de Chimie de la Matière Condensée de Paris (LCMCP), UMR 7574, 4 place Jussieu, 75005 Paris, France Email Address: cedric.boissiere@upmc.fr
Michel Wong Chi Man
Institut Charles Gerhardt Montpellier, Univ. Montpellier, CNRS, ENSCM, 34293 Montpellier, France
Jakub Kusz
École Normale Supérieure de Lyon, CNRS, Université Claude Bernard Lyon 1, Laboratoire de Chimie, UMR 5182, 46 Allée d'Italie, 69364 Lyon, France
Colloid Chemistry Department, Max Planck Institute of Colloids and Interfaces, 14476 Potsdam, Germany

Position [cm^{-1}]	Intensity	Assignment
1696	s	μ (C=O) free
1639	s	μ (C=O) HB
1623	m	μ (C-C) in aryl coupled to δ N−H
1605	m	
1593	s	
1563	s	δ (N−H)
1540	m	
1518	w	
1501	m	ip aryl μ (C-C) and δ (C−H)
1488	m	δ (N−H)

Table S1: FT-IR assignment of PhU-TES in the amide I and amide II regions based on DFT calculation and literature data[1, 2]. w - weak, m - medium, s - strong; δ - bending, μ - stretching.

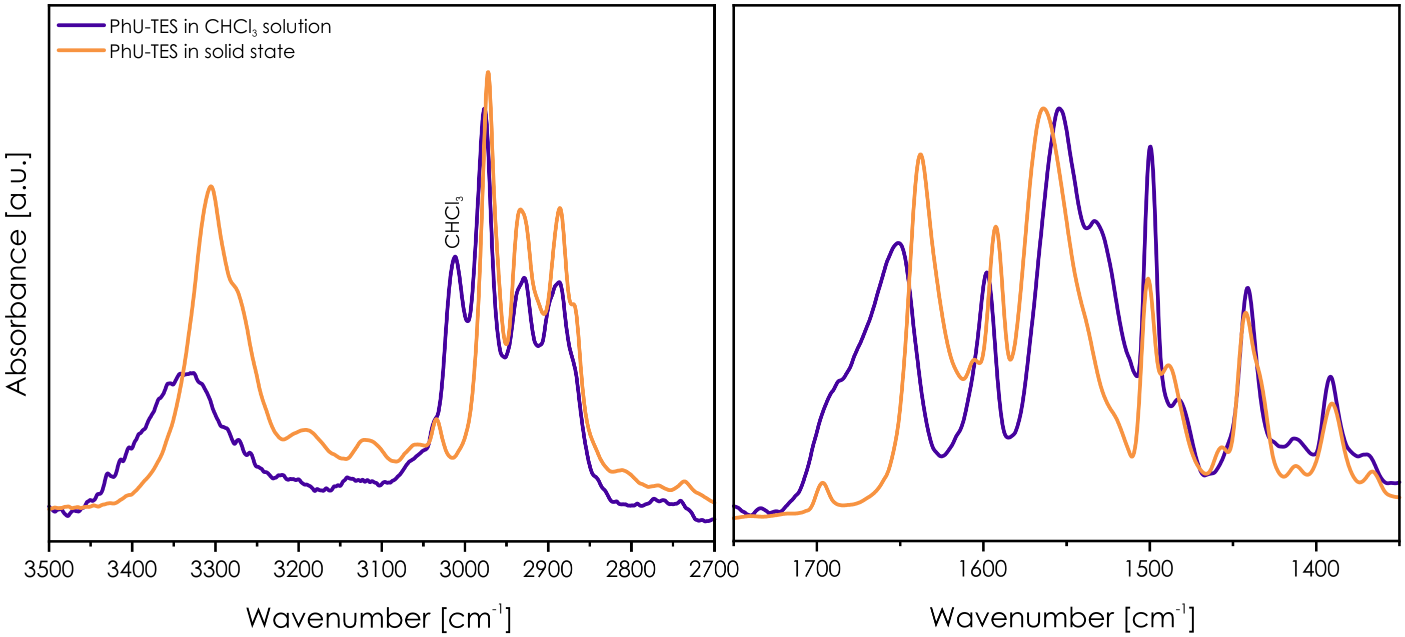

Figure S1: Evolution of IR spectra for PhU-TES precursor after dilution in $CHCl_3$.

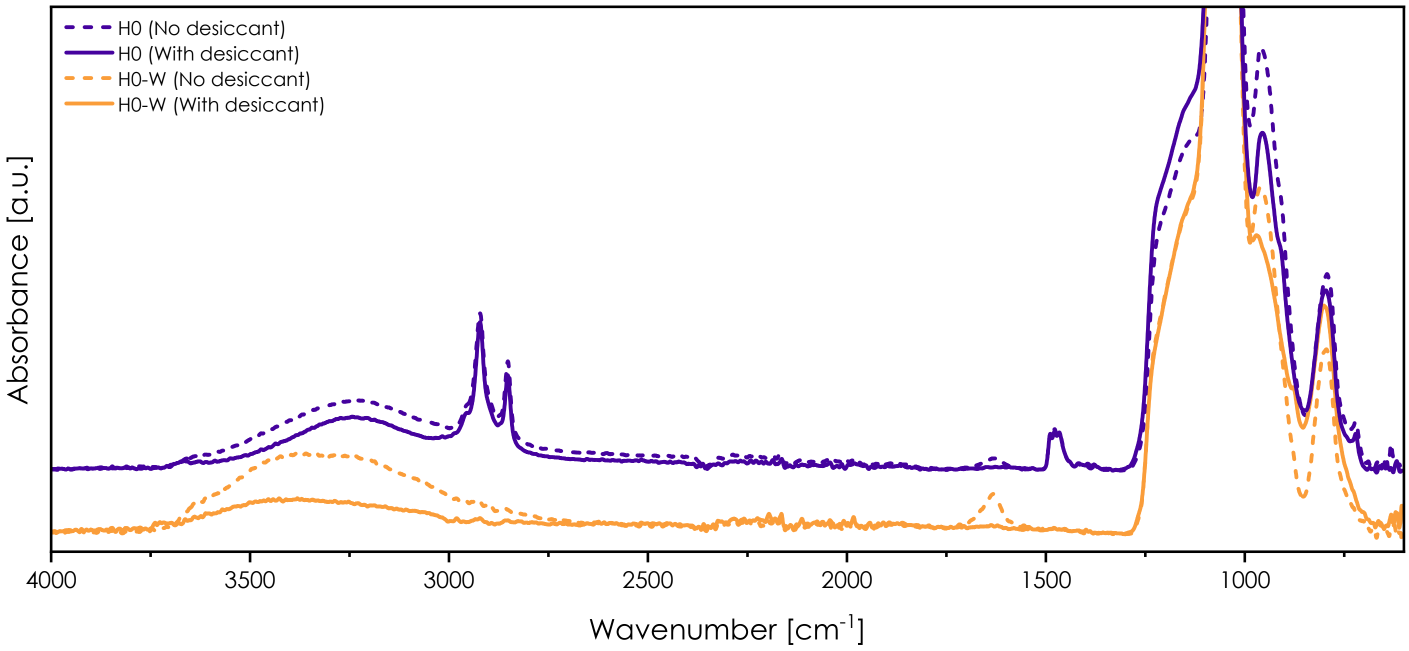

Figure S2: IR spectra of H0 and H0-W films recorded with and without the presence of the desiccant.

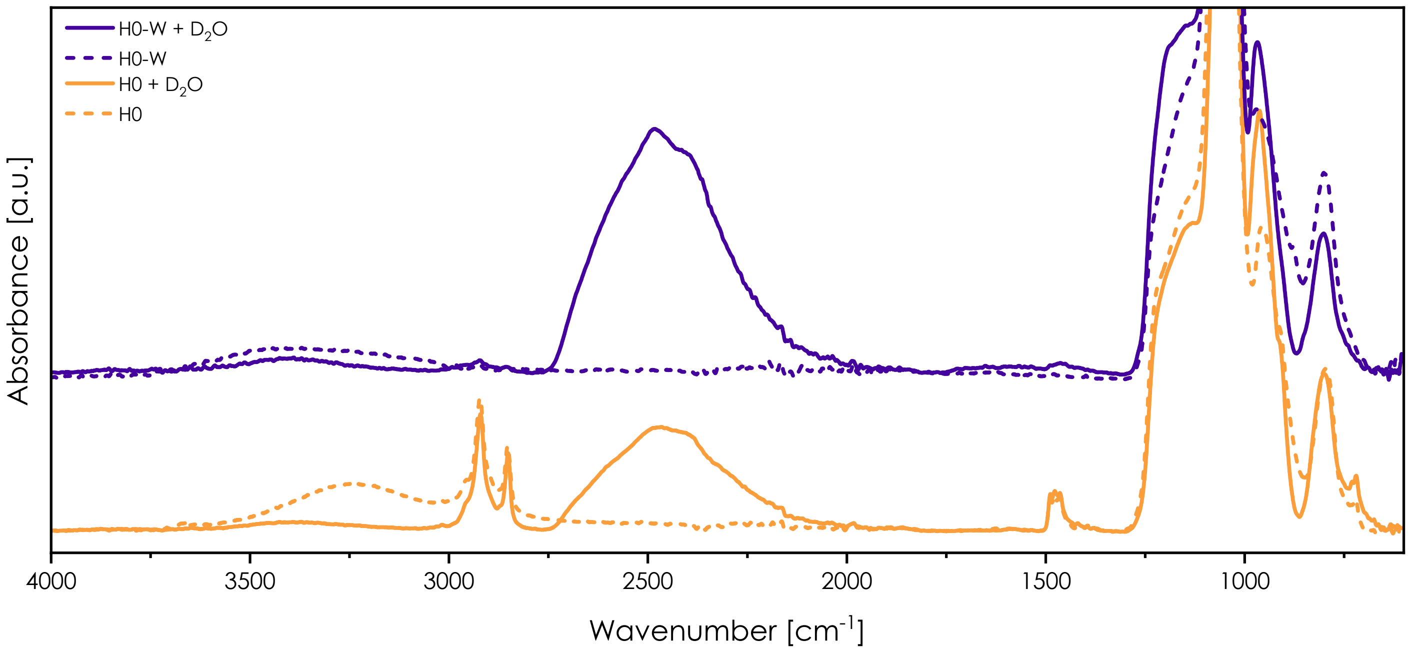

Figure S3: IR spectra of H0 and H0-W films recorded with and without the presence of the deuterium oxide vapors.

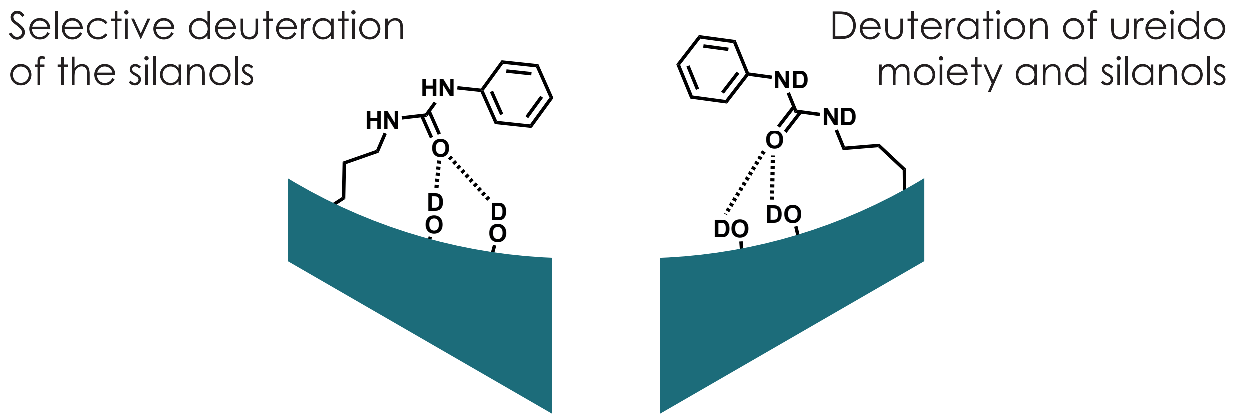

Figure S4: Schematic view on the deuterium exchange in hybrid samples. Selective deuteration (left scheme) seems to be an improbable mechanism. We should expect rather a global deuteration of silanols and ureido groups (right scheme).

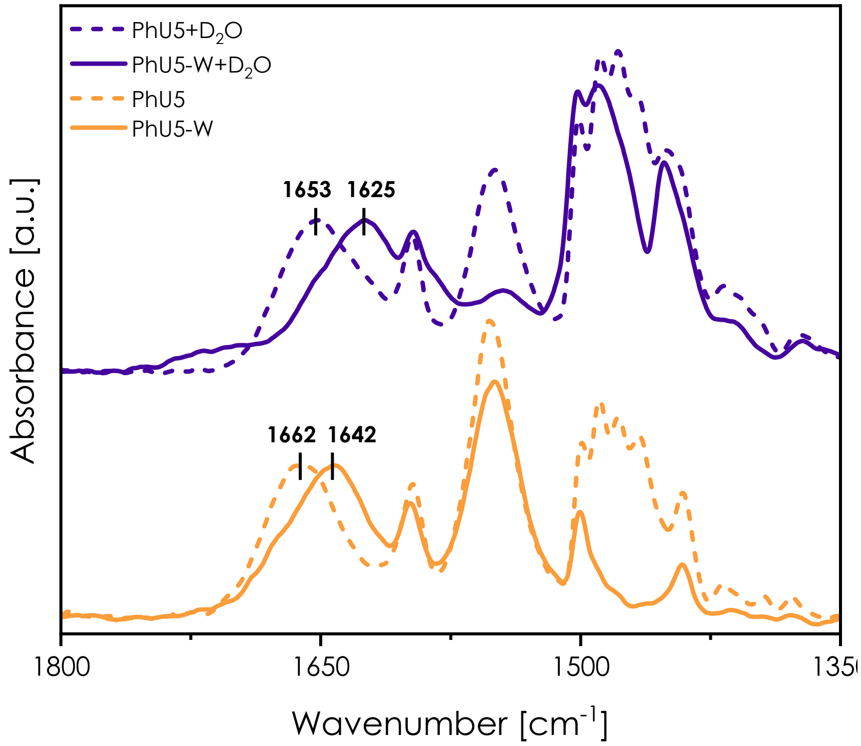

Figure S5: Infrared spectra of PhU5 and PhU5-W hybrid films before and after the H-D exchange.

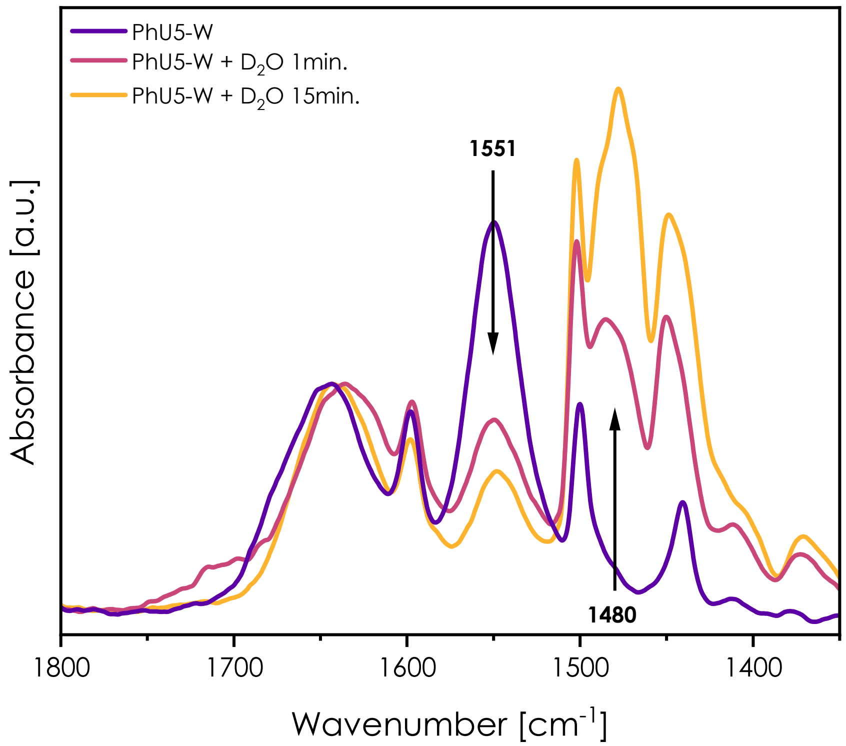

Figure S6: Infrared analysis of the deuterium exchange performed on hybrid, porous films. H-D exchange is not an immediate process and occurs on a timescale of several minutes.

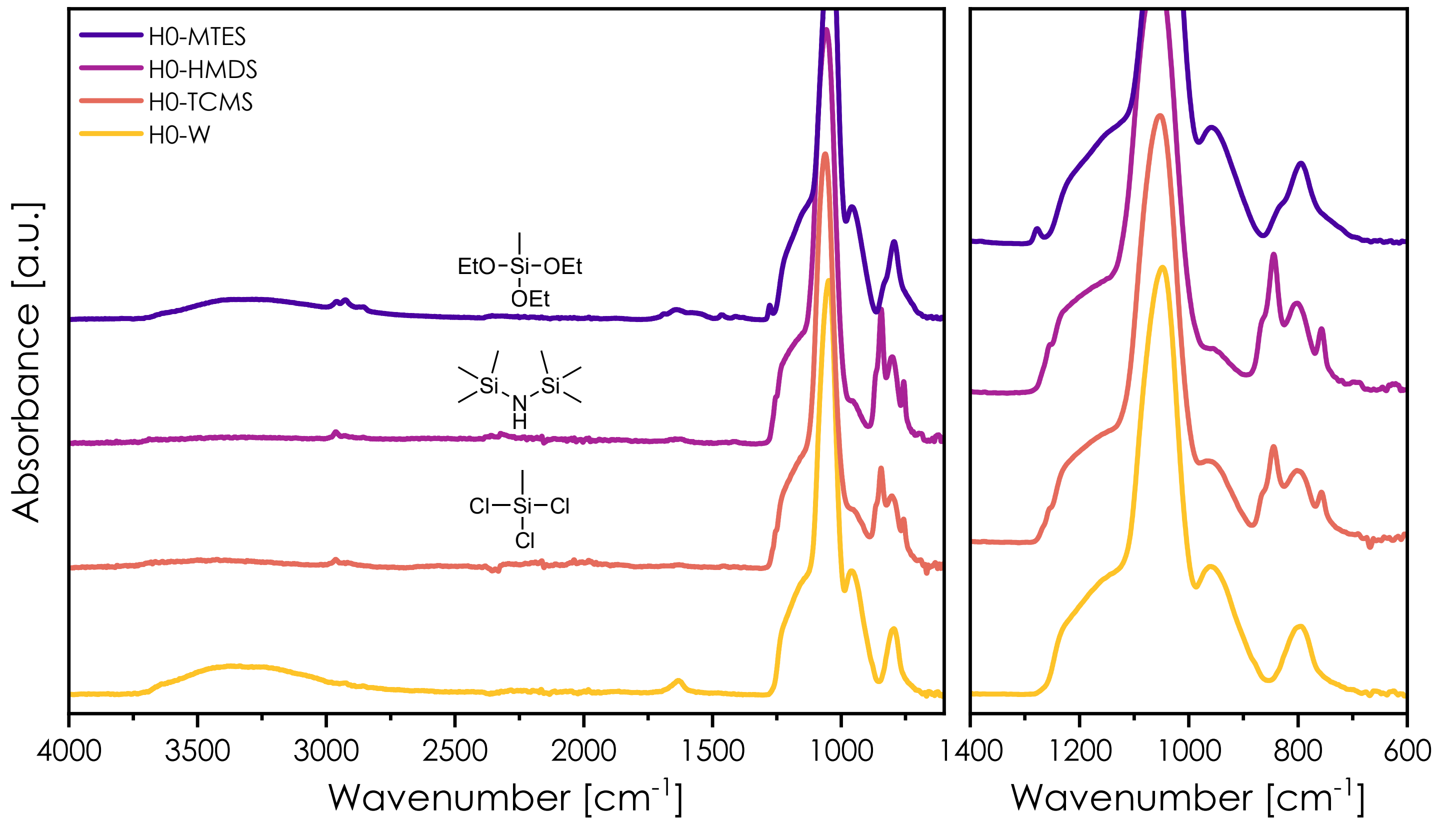

Figure S7: Infrared spectra of referential H0 films hydrophobized with different agents: From the top: co-condensation with MTES, post-functionalization with HMDS, post-functionalization with TCMS, non-hybrid film.

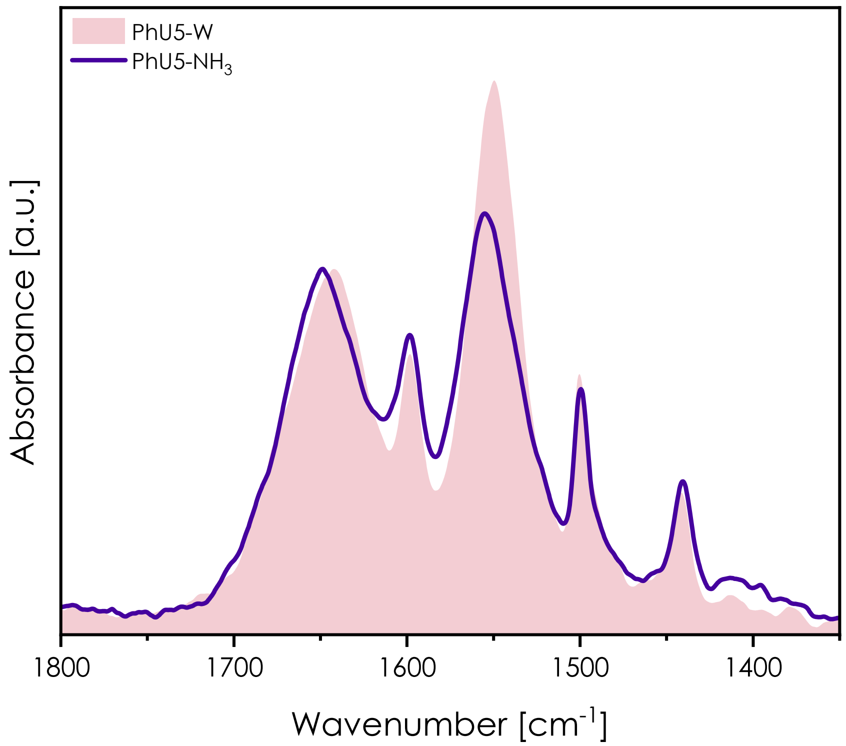

Figure S8: Amide I and II regions in the IR spectrum of PhU5-NH_3 film that was treated with ammonia vapors. For comparison, the spectrum of the PhU5-W sample is given in the background.

To achieve exclusively silanol condensation, without inducing steric effects we exposed the film to NH_3 vapor, which is known to condense available silanols[3]. We prepared a sample PhU5-NH3 by keeping the film in a sealed chamber containing an aqueous ammonium hydroxide solution (28%). Unfortunately, the reduction of the μ(Si-OH) band was not satisfactory compared to other methods. This effect may be due to HB formed between the ureido and silanol groups, which hinder the condensation of silanol. However in Figure S8, we observe small upshifts of amide I (Δ_I=7 cm^{-1}) and amide II bands (Δ_{II}=5 cm^{-1}), which could be explained by replacing a fraction of SiOH-U bonds with U-U dimers.

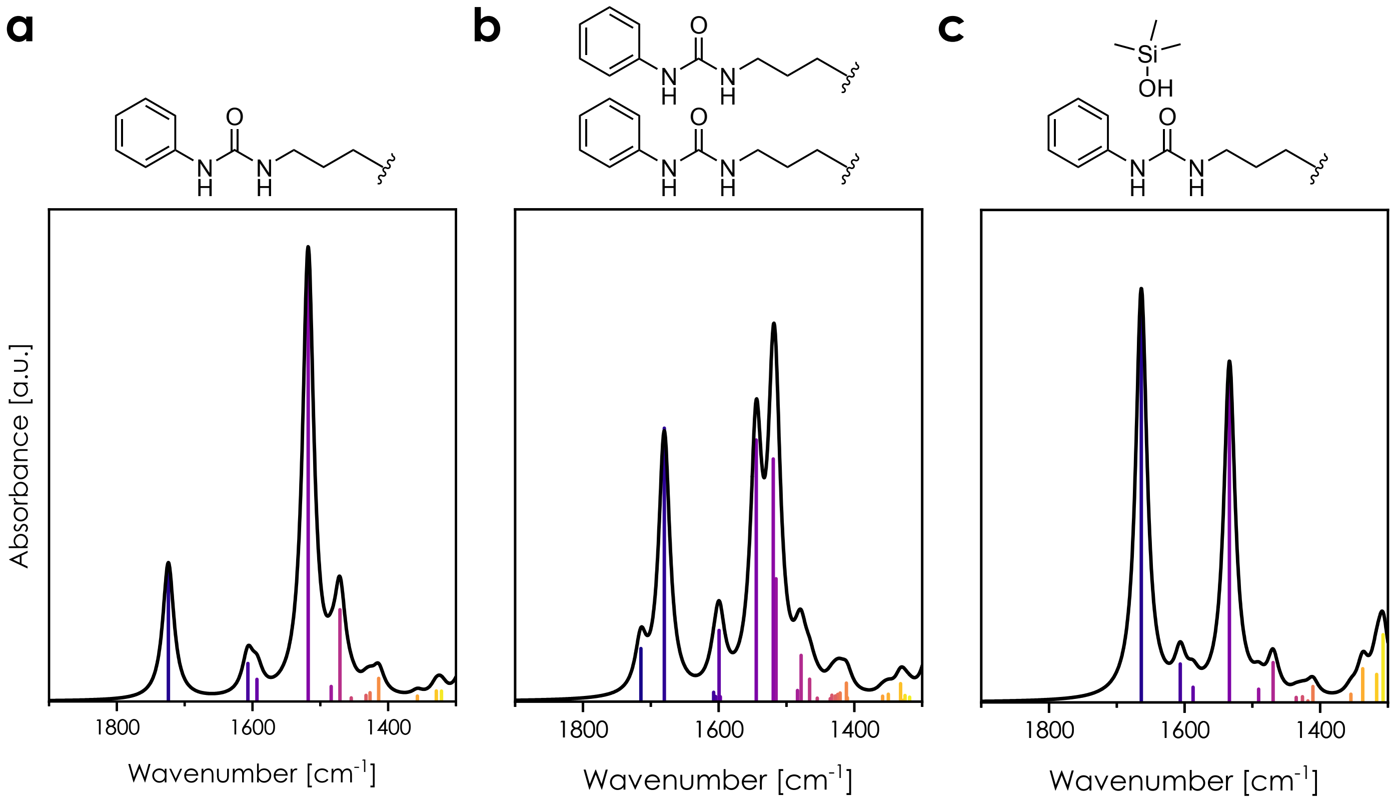

Figure S9: Theoretical (DFT) spectra obtained for free ureido groups, ureido-ureido dimer, and ureido-silanol bonding.

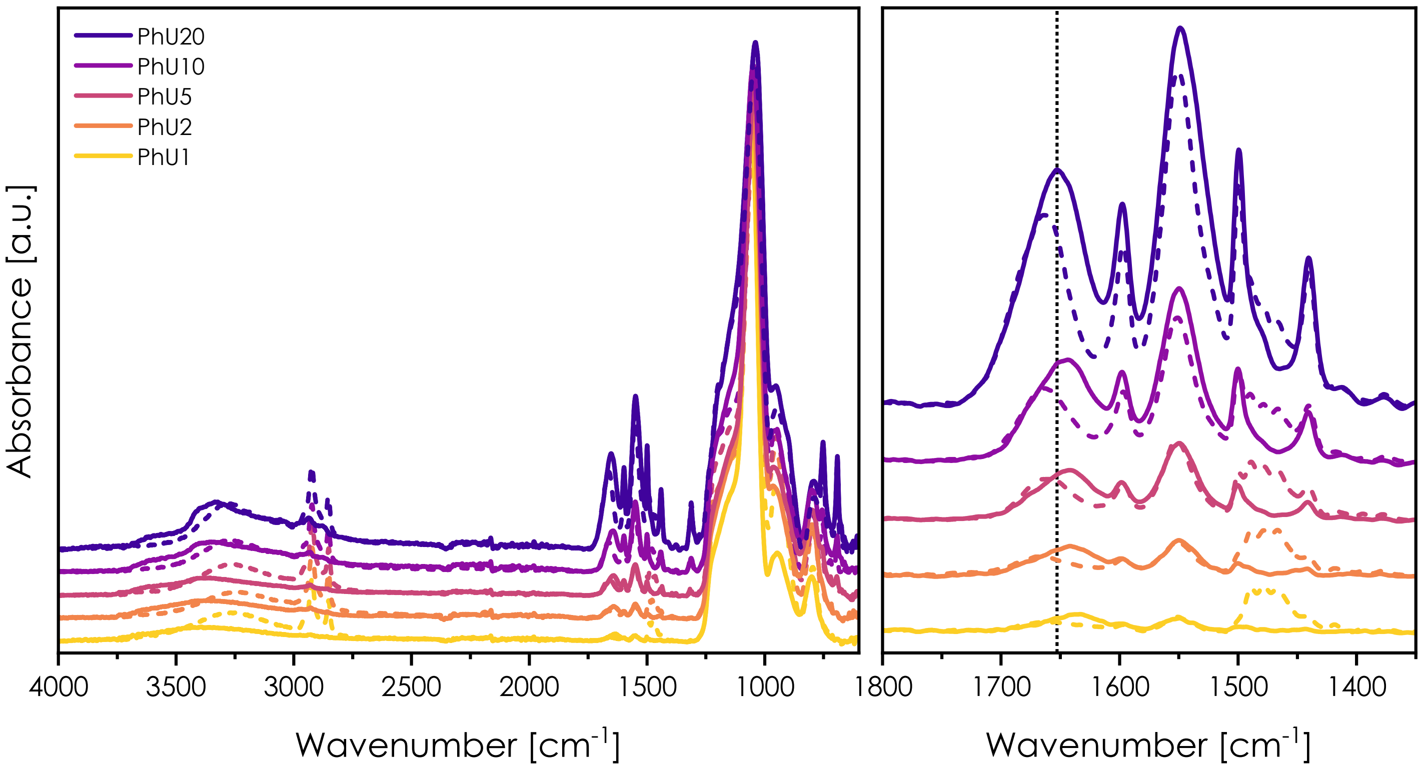

Figure S10: Full spectra of PhU1-PhU20 films normalized to (Si-O-Si) vibrational band. Only very weak signals of the amide I and amide II bands were obtained for PhU2 and PhU1 films. Dashed line corresponds to peak of amide I band for PhU20-W sample.

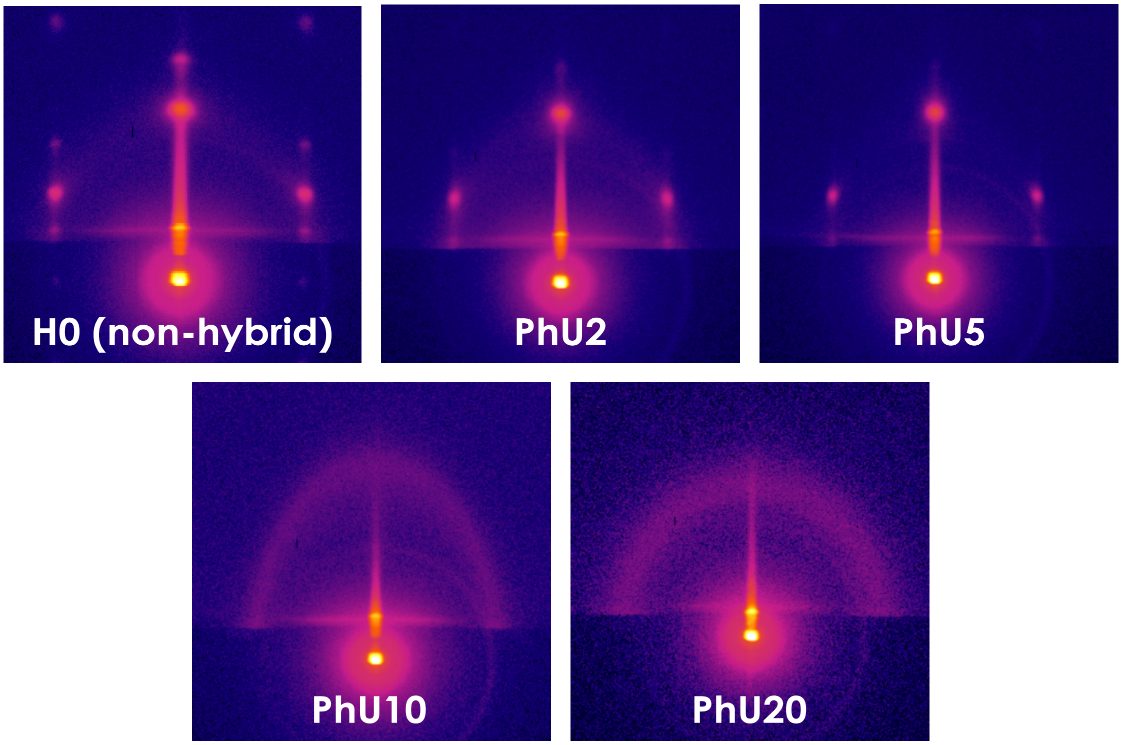

Figure S11: GI-SAXS patterns of non-hybrid and hybrid thin films.

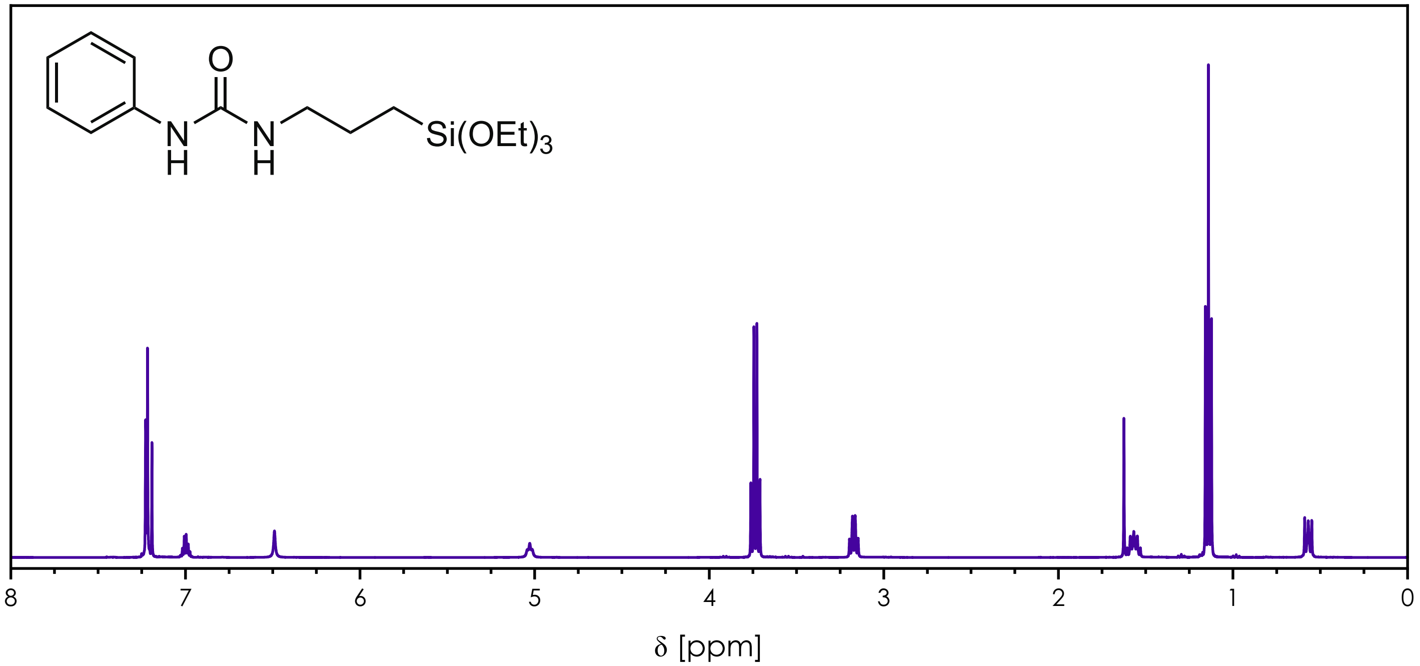

Figure S12: ^{1}H NMR spectrum of PhU-TES precursor.

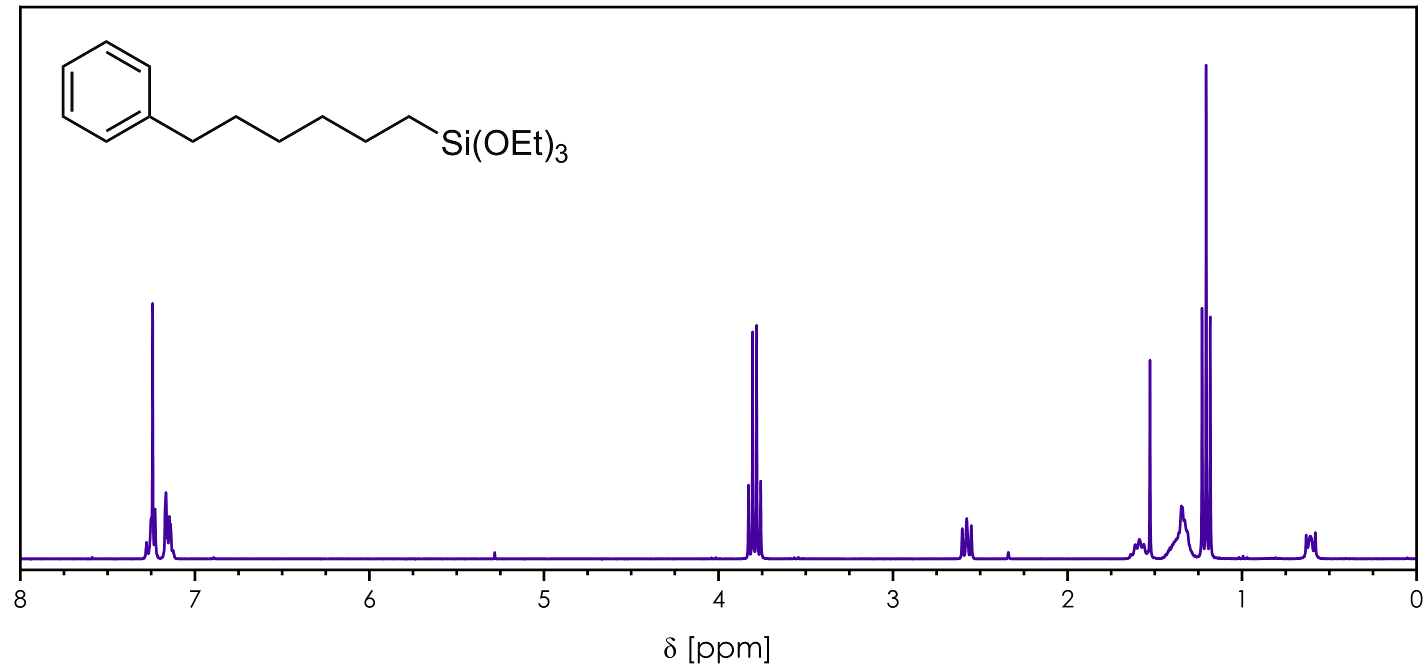

Figure S13: ^{1}H NMR spectrum of PhHex-TES precursor.

References

[1] R. Le Parc, V. T. Freitas, P. Hermet, A. M. Cojocariu, X. Cattoen, H. Wadepohl, D. Maurin, C. H. Tse, J. R. Bartlett, R. A. S. Ferreira, L. D. Carlos, M. Wong Chi Man, J. L. Bantignies, Infrared and Raman spectroscopy of non-conventional hydrogen bonding between N,N'-disubstituted urea and thiourea groups: a combined experimental and theoretical investigation, *Phys. Chem. Chem. Phys.* **2019**, *21*, 6 3310.

[2] G. Creff, Thesis, Université Montpellier II, **2012**.

[3] M. Boudot, V. Gaud, M. Louarn, M. Selmane, D. Grosso, Sol–Gel Based Hydrophobic Antireflective Coatings on Organic Substrates: A Detailed Investigation of Ammonia Vapor Treatment (AVT), *Chem. Mater.* **2014**, *26*, 5 1822.
